# Species‐complex diversification and host‐plant associations in *Bemisia tabaci*: A plant‐defence, detoxification perspective revealed by RNA‐Seq analyses

**DOI:** 10.1111/mec.14865

**Published:** 2018-10-10

**Authors:** Osnat Malka, Diego Santos‐Garcia, Ester Feldmesser, Elad Sharon, Renate Krause‐Sakate, Hélène Delatte, Sharon van Brunschot, Mitulkumar Patel, Paul Visendi, Habibu Mugerwa, Susan Seal, John Colvin, Shai Morin

**Affiliations:** ^1^ Department of Entomology The Hebrew University of Jerusalem Rehovot Israel; ^2^ Department of Biological Services Weizmann Institute of Science Rehovot Israel; ^3^ Department of Plant Protection School of Agriculture São Paulo State University Botucatu Brazil; ^4^ CIRAD‐UMR PVBMT Saint Pierre, La Réunion France; ^5^ Natural Resources Institute University of Greenwich Kent UK; ^6^ School of Biological Sciences The University of Queensland Brisbane Queensland Australia

**Keywords:** *Bemisia tabaci*, diet breadth, diversification, host adaptation, insect–plant interactions, species complex

## Abstract

Insect–plant associations and their role in diversification are mostly studied in specialists. Here, we aimed to identify macroevolution patterns in the relationships between generalists and their host plants that have the potential to promote diversification. We focused on the *Bemisia tabaci* species complex containing more than 35 cryptic species. Mechanisms for explaining this impressive diversification have focused so far on allopatric forces that assume a common, broad, host range. We conducted a literature survey which indicated that species in the complex differ in their host range, with only few showing a truly broad one. We then selected six species, representing different phylogenetic groups and documented host ranges. We tested whether differences in the species expression profiles of detoxification genes are shaped more by their phylogenetic relationships or by their ability to successfully utilize multiple hosts, including novel ones. Performance assays divided the six species into two groups of three, one showing higher performance on various hosts than the other (the lower performance group). The same grouping pattern appeared when the species were clustered according to their expression profiles. Only species placed in the lower performance group showed a tendency to lower the expression of multiple genes. Taken together, these findings bring evidence for the existence of a common detoxification “machinery,” shared between species that can perform well on multiple hosts. We raise the possibility that this “machinery” might have played a passive role in the diversification of the complex, by allowing successful migration to new/novel environments, leading, in some cases, to fragmentation and speciation.

## 1 INTRODUCTION

Species complexes, including cryptic ones, are present in a wide range of taxonomic groups and are being discovered at an increasing rate (Bickford et al., 2007). They provide an excellent tool for connecting the study of taxonomy and phylogenetic patterns with ecosystems functioning and evolutionary processes such as speciation (Struck et al., 2017), giving insights not only into the establishment of reproductive barriers between the diverging species (Rundle & Nosil, 2005), but also into functional changes occurring in their genomes (Simon et al., 2015).

Species complexes of herbivorous insects are of special interest, because host specialization is the favoured and dominant evolutionary strategy in this insect group (Forister et al., 2015). It is to be expected, therefore, that within species complexes of herbivorous insects, most species will be specialists or oligophages, with the exception of a few “true” generalists (Loxdale & Harvey, 2016). The traditional assumptions argue that specialism predominates because it allows host‐specialized herbivores to become optimally adapted to the nutritional and secondary defensive chemistry of their host plants (Cornell & Hawkins, 2003), while generalism is adaptive mostly when the availability of high‐quality host plants is unpredictably variable (Forister, Dyer, Singer, Stireman, & Lill, 2012). Recently, however, Forister and Jenkins (2017) demonstrated that communities enriched in specialized species (relative to generalized taxa) can evolve in the absence of genotype‐by‐environment interactions that confer a direct advantage to the specialists over the generalists. Alternatively, random diversification forces acting on fragmented populations over geographical ranges are sufficient for producing multiple speciation events. In most cases, examination of such closely related species groups is considered to be the best experimental approach for studying the evolution of different degrees of generalism or specialism and their putative derived effects on divergence and speciation processes (Forister et al., 2012; Nylin & Janz, 2009; Nyman, 2010). This is because species complexes tend to share a common genetic background, with biological differences mainly associated with their feeding ecology, thus avoiding the “phylogenetic noise” present when comparing phylogenetically distant taxa that have accumulated more diverse adaptations (Roy et al., 2016).

We focus here on the whitefly, *Bemisia tabaci* (Hemiptera: Aleyrodidae) species complex, which is a cosmopolitan complex widely distributed throughout tropical and subtropical regions. Until about 20 years ago, *B. tabaci* was considered as a highly variable species comprising a series of morphologically indistinguishable biotypes that differ mostly in fecundity, insecticide resistance and the capability of transmission of viruses (Brown, Frohlich, & Rosell, 1995). However, multiple studies (mostly phylogenetic analyses and crossing experiments) came to the conclusion that it might be more accurate to regard *B. tabaci* as a cryptic species complex, rather than a highly variable species (De Barro, Liu, Boykin, & Dinsdale, 2011; Dinsdale, Cook, Riginos, Buckley, & De Barro, 2010; Liu, Colvin, & De Barro, 2012). Dinsdale et al. (2010) proposed a general threshold of 3.5% for mitochondrial cytochrome oxidase I (mtCOI) DNA sequence divergence for species delimitation, which currently leads to the identification of at least 35 distinct species assigned to ~11 major clades (Barbosa et al., 2014; Hu et al., 2014). With few minor non‐conclusive exceptions, reproductive compatibilities’ experiments confirm so far the accuracy of the cryptic species concept adding credibility to the mtCOI approach (Liu et al., 2012; Qin, Pan, & Liu, 2016).

The understanding of the mechanism(s) driving the extreme and unusual diversification of the *B. tabaci* species complex is limited. Explanations provided so far largely point towards geographical (allopatric) divergence as the key driving force, associated with the separation of continental landmasses, which overlapped with a period of global diversification across the plant and animal kingdoms (Boykin, Bell, Evans, Small, & De Barro, 2013; De Barro, Trueman, & Frohlich, 2005). These arguments were based on the association of different species with particular geographical (continental) regions, the lack/minimal of gene flow between species and the assumption that most species share a similar and broad host range (De Barro, 2005). However, our knowledge of the host‐plant range of the different species is patchy. Moreover, the allopatric divergence model by itself might fail to explain prominent within continental expansions in the complex, as observed, for example, in the Asia II (~12 species) or sub‐Saharan Africa (~13 species) major genetic groups (Lee, Park, Lee, Lee, & Akimoto, 2013; Mugerwa et al., 2018).

Here, we explored the possibility that evolved differences in host utilization could have played a role in the diversification of the *B. tabaci* species complex. Our major goal was to identify macroevolution changes in the relationship between *B. tabaci* species and their host plants that have the potential to promote diversification. At first, we tested the hypothesis that the species complex is a more host‐specific taxon than commonly thought. Specifically, we hypothesized that species in the complex will vary not only in the hosts they actually use, as this might reflect the hosts that are present in a specific geographical area, but also in their host range (Brown et al., 1995; Perring, 2001). We expected, however, that the latter will show more of a quantitative effect (significant differences in performance on hosts that have been lost by some species) than a qualitative one (cases in which some hosts cannot be utilized at all by some species), as previously demonstrated in few studies (De Barro & Bourne, 2010; Iida, Kitamura, & Honda, 2009; Xu, Lin, & Liu, 2011). Host‐associated differentiation was shown to play an important role in speciation of various nongeneralist phytophagous insects (Berlocher & Feder, 2002; Stireman, Nason, & Heard, 2005). However, evidence for the putative importance of the phenomenon in generalist species is also accumulating. For example, the polyphagous aphid *Myzus persicae* comprises a specialized form on tobacco that is formally designated as the subspecies *M. persicae nicotianae* (Margaritopoulos, Malarky, Tsitsipis, & Blackman, 2007). Other examples include grasshoppers and green mirids, which feed on multiple hosts from different families, yet were shown to exhibit host‐associated differentiation (Antwi, Sword, & Medina, 2015). These cases of host‐associated differentiation were shown to maintain gene flow across large distances (Dermauw, Pym, Bass, Van Leeuwen, & Feyereisen, 2018; Hereward, Walter, DeBarro, Lowe, & Riginos, 2013).

We then focused on one important component (in herbivory) of successful plant feeding, the ability to counteract (detoxify) the toxic effect of plant‐chemical defences (Celorio‐Mancera et al., 2013; Dermauw et al., 2013; Heckel, 2014; Ragland et al., 2015; Wybouw et al., 2015). The detoxification system works in three phases: The first phase involves oxidation, hydrolysis and/or reduction by enzymes such as P450 monooxygenases (P450s) and carboxylesterases (COEs); the second phase involves conjugation with hydrophilic groups such as glutathione, sulphate or sugars by glutathione S‐transferases (GSTs), sulfotransferases or UDP‐glucosyltransferases (UDPGTs) to increase polarity and facilitate excretion; and the third phase involves active export of the conjugated toxins out of the cell by ATP‐binding cassette transporters (ABC transporters; Després, David, & Gallet, 2007). Multiple studies have indicated that, in insect herbivores, an extensive re‐arrangement of detoxification gene expression (transcriptional plasticity) takes place shortly after host shifts, clearly suggesting that the detoxification system plays an important role in the insects’ survival, when first encountering a novel or adverse host plant (Celorio‐Mancera et al., 2013; Grbić et al., 2011; Matzkin, 2012; Yu, Fang, Zhang, & Jiggins, 2016). Moreover, it was argued that selection on the mode of expression of these genes could be disproportionately strong (Wybouw et al., 2015) as insect herbivores may use the transcriptional plasticity displayed upon the first exposure to the novel host, not only to secure their initial survival (phenotypic accommodation) but also to facilitate subsequent adaptation (Nylin & Janz, 2009; Schlichting & Wund, 2014). If inclusion of the novel host in the normal range of hosts is important enough for fitness, plastic traits may undergo genetic accommodation by acquiring quantitative genetic changes that can either increase or decrease their environmental responsiveness (Levis & Pfennig, 2016). In some cases, selection can cause a plastic trait to lose its environmental responsiveness which can result in the constitutive expression of genes (Schneider & Meyer, 2017). Constitutive expression levels can allow the refinement of the expression levels (via selection), for obtaining optimal performance that outcompetes that obtained by the ancestral plastic trait (Levis & Pfennig, 2016; Pfennig & Ehrenreich, 2014; Wang et al., 2017). We therefore expected that both plastic and/or constitutive expression differences in detoxification genes will be present between closely related species that differ in their host range (Ragland et al., 2015; Roy et al., 2016; Wybouw et al., 2015).

We hypothesized that, during evolution, various species in the *B. tabaci* complex have acquired genetic changes that relate to their ability or inability to utilize plant hosts. Therefore, we asked if the expression profile of the insect's detoxification system is shaped more by phylogenetic constraints or by differences in the ability to perform well in heterogeneous environments containing multiple suitable and novel plant hosts. We hypothesized that, if genetically more similar species would show more similar expression patterns, it would suggest that random genetic drift and/or phylogenetic constraints have shaped the detoxification gene expression evolution within each clade. This finding would preclude us from drawing a link between differences in the species performance on various host and speciation as the former might only be a consequence of reproductive isolation rather than causing it (Peccoud et al., 2010). Our alternative hypothesis was that the detoxification gene expression patterns will discriminate between species showing high performance on multiple hosts and species not capable of performing well on multiple hosts (more or less specialized species, respectively). More precisely, we hypothesized that species that might be only distantly related in the phylogenetic tree, but are capable of utilizing successfully multiple hosts, will be found to share common detoxification patterns (mechanisms) either by maintaining an ancestral expression state or by convergent evolution that allow to cope with a wide variety of plant‐chemical defences. This can support a species diversification and expansion model in which the general expression pattern facilitates larger geographical distributions and/or survival in new and/or novel environments (plant hosts), which might lead to fragmentation and eventually speciation due to both random (drift) and adaptive (selection) processes (Forister et al., 2012; Janz & Nylin, 2008; Nylin & Janz, 2009; Nyman, 2010). Evidence that such processes could occur was recently presented in three polyphagous and widespread lepidopteran species: the brown tail moth *Euproctis chrysorrhoea* (Marques, Wang, Svensson, Frago, & Anderbrant, 2014), the gypsy moth *Lymantria dispar* (Lazarević et al., 2017) and the tussock moths taxon (subfamily Lymantriinae; Wang et al., 2017).

In order to test our hypothesis, we first conducted a literature survey in which we documented the sampled host range of the various species in the *B. tabaci* complex. Based on the literature survey findings, we selected six *B. tabaci* species that represent different geographical and documented host‐range groups and four host plants: a common host naturally shared by all species and three noncommon and putatively toxic hosts that are utilized only by some species in the complex. Next, we obtained the performance and transcriptome profiles of the six *B. tabaci* species during host shifts from the common host to the three noncommon hosts. Gene expression across species and plants was then assessed to determine whether variation was explained better by the species performance on the four hosts or by the phylogenetic relatedness among the six *B. tabaci* species.

## 2 METHODS

### 2.1 Literature survey, clustering and ancestral host reconstruction analysis

The literature survey was conducted by typing the terms (using Boolean operators): “*Bemisia tabaci*” AND botanical family name (e.g., “Acanthaceae”) AND “Cytochrome oxidase I” OR “mtCOI” (both options) in Google Scholar, using the “Any time” option. Only reported insect collections in which the plant host was associated with a specific mtCOI barcode were maintained (papers are listed in Supporting Information Table S1). Due to too sporadic sampling reports, data of species within the Mediterranean (MED), New‐World (NW), Sub‐Saharan Africa (SSA), Italy, Asia II and China genetic groups were collapsed. The combined data set (the absence/presence matrix) was used as input for the heatmap.2 function (Euclidean distance with a complete linkage method) of the r gplots package (R Core Team, 2017).

For ancestral host‐range reconstruction (order, family and genus levels), a maximum likelihood (ML) inferred tree, for all major genetic groups of *B. tabaci* and two related outgroups (*Bemisia afer* and *Dialeurodes citri*), was first produced (Supporting Information Figure S1a). The mtCOI nucleotide sequences were downloaded from the genbank database, clustered with CD‐HIT (Fu, Niu, Zhu, Wu, & Li, 2012) at 98% identity, and a cluster representative for each major group was selected (in case where more than one cluster was obtained, a single representative was selected). A codon‐based alignment was performed with the revtrans2.0 web server (Wernersson & Pedersen, 2003), and iq‐tree (Nguyen, Schmidt, von Haeseler, & Minh, 2014) was used to calculate the best codon model (MGK+F3X4+R2) and ML tree (5,000 ultrafast bootstraps and 5,000 SH‐aLRT). The host plant ranges of the last common ancestors (LCAs) of the different *B. tabaci* species were estimated with the ace function (type = “discrete,” method = “ML,” CI = T, marginal = F) in the ape package (Paradis et al., 2004) from r using the information from Supporting Information Table S1. The resulting tree, together with the corresponding presence/absence matrix of order or genus host usage, was used for the ML (marginal) and maximum parsimony (MP) ancestral states reconstruction, using the ace (equal rate) and MPR functions of the ape package, respectively (r software).

### 2.2 *Bemisia tabaci* and host plant species

Six species of *B. tabaci*, representing different geographical and diet‐breadth groups, were selected for analyses: SSA1‐SG3 (Sub‐Saharan Africa genetic group 1, sub‐group 3, collected in Tanzania in 2013/maintained on *Manihot esculenta*), Asia II‐1 (Asia‐II genetic group, species 1, collected in Pakistan in 2013/maintained on *Gossypium hirsutum*), New‐World 2 (hence after NW2) (New‐World genetic group, species 2, collected in Brazil in 2013/maintained on *Solanum lycopersicum*), and MEAM1 (Middle East‐Asia Minor species 1), MED‐Q1 (Mediterranean Q species 1) and Uganda‐MED‐ASL (Mediterranean nonsilverleafing sub‐group from Uganda) (Africa/Middle East/Asia minor genetic group, collected in Peru in 2012/maintained o *Gossypium hirsutum*; France in 2011/maintained on *Capsicum annuum* and Uganda in 2012/maintained on *Ipomoea batatas*, respectively). The identity of the six species was verified using their mtCOI DNA sequences (deposited in study accession number PRJEB21948). At least 2 months (~3–4 generations) before starting the experiments, ~500 founders from each of the six colonies were transferred to eggplant, to allow them to establish a common baseline host plant. Colonies were reared under standard conditions of 28 ± 2°C, 60% humidity and a 14:10‐hr light:dark cycle.

The selection of the experimental host plants was based on the results of the literature survey and the host reconstruction analysis, which identified common host plants, shared by many *B. tabaci* species, and noncommon host plants, that are utilized by only few species. Based on this, four host plants were selected, eggplant, a common host (*Solanum melongena*, cv. Black Beauty, Solanaceae/Solanales), and three noncommon host plants, also known to produce toxic phytotoxins: pepper (*Capsicum annuum*, cv. California Wonder; Solanaceae/Solanales), cassava (*Manihot esculenta*, cv. MCol22; Euphorbiaceae/Malpighiales) and kale (*Brassica oleracea*, var. *sabellica*, cv. Dwarf Green Curled; Brassicaceae/Brassicales). The Solanaceae/Solanales seems to be one of the ancient host families/orders of *B. tabaci*, common to many species in the complex (Supporting Information Figure S1b), but this observation relates mostly to the *Solanum* genus. Moreover, differences in the probability of being part of the ancestral host repertoire of *B. tabaci* were observed between the *Solanum* and *Capsicum* genera (*p *=* *0.98 and *p *=* *0.5, respectively; Supporting Information Figure S1c). Toxic defensive secondary metabolites that are known to be present in pepper include flavonoids, phenols and capsaicinoids (Mokhtar et al., 2015). Like the Solanaceae/Solanales, the Euphorbiaceae/Malpighiales seem to be one of the ancient plant host families/orders of *B. tabaci*, common to many species in the complex (Supporting Information Figure S1d). However, cassava is considered to be a well‐defended plant and a suitable host only for some SSA and Asia II species of *B. tabaci* (Colvin, Omongo, Maruthi, Otim‐Nape, & Thresh, 2004; Ellango et al., 2015). Important defensive metabolites present in cassava include cyanogenic glucosides (Alves, 2002) and flavonoids (Prawat et al., 1995). Unlike the Solanaceae/Solanales and the Euphorbiaceae/Malpighiales, Brassicaceae/Brassicales plants are not utilized by many species in the *B. tabaci* complex. Moreover, the probability of the family/order to be part of the ancestral host repertoire of *B. tabaci* was estimated to be only 0.5 (Supporting Information Figure S1e). According to the literature survey, species within the *B. tabaci* complex mainly utilize host plants from one tribe, the Brassicaceae. Important toxic metabolites present in kale include glucosinolates and flavonoids (Schmidt et al., 2010). All experimental plants were grown in rearing rooms maintained at 28 ± 2°C, 60% humidity and a14:10 ‐hr light:dark cycle.

### 2.3 Performance assay

Groups of 50 *B. tabaci* adults, from each of the six species reared on eggplant, were transferred 1–3 days after emergence, to one of the four host plants (eggplant, pepper, kale and cassava), at the 5‐ to 8‐leaf stage. The adults fed on the four host plants for 24 hr, after which the proportion of survivors was recorded. Proportional data were arcsin‐square root transformed. Two‐way ANOVA followed by sequential Bonferroni comparisons, using the conservative Dunn–Sidak method (Sokal & Rohlf, 1995), was carried out to compare the mean survival rate of the six species on the four plant hosts.

### 2.4 Establishing a detoxification data set for *B. tabaci*

Raw data from several *B. tabaci* transcriptomes were downloaded: MEAM1: SRX022878, SRA036954 and SRR835757; MED‐Q1: SRX018661, SRR316271, SRR835756 and PRJNA293094; and Asia II‐3: SRR062575. Transcriptomes were assembled with trinity v2.0.6 adapted to pair‐end libraries (with the exception of SRA036954, which was assembled as single end) and with the following options “trimmomatic” and “normalize_reads” activated. Re‐assembled transcriptomes were deposited in an in‐house Galaxy server to perform the following steps. The sequences were clustered by CD‐HIT‐EST at 95% identity, considering the sequences belonging to the same cluster as allelic variants. For sequence annotation, a blastx similarity search against the ncbi protein database nr (e‐value threshold 10^−6^) was performed, keeping the accession number of the top hit in the insect model species: *Drosophila melanogaster*,* Helicoverpa armigera* and *Acyrthosiphon pisum* (Supporting Information Table S2). This process allowed us to produce a non‐redundant detoxification consensus gene data set containing 104 P450s, 25 GSTs, 24 COEs, 71 UDPGTs, 20 sulfotransferases and 54 ABC transporters.

### 2.5 RNA isolation and Illumina sequencing

Groups of 200 newly emerged adults from each species, grown on eggplant, were subjected to a feeding period of 72 hr on 10% sucrose diet, to obtain a standardized detoxification gene expression pattern, which was host plant‐independent. The groups were then transferred for a feeding period of 24 hr, to the four experimental host plants: eggplant, pepper, kale and cassava. Next, the surviving adults were collected for RNA extraction. About 50 adults were pooled for each RNA sample to obtain sufficient RNA. Three feeding experiments per host plant were performed for each *B. tabaci* species. Total RNA was extracted according to the manufacturer's instructions (Isolate II mini kit, Bioline). Library construction and sequencing were performed by the Centre for Genomic Technologies at the Hebrew University of Jerusalem, using a NextSeq 500 desktop sequencer, which produced approximately 27 million 75‐bp single‐end reads per sample.

### 2.6 Gene expression analysis

The reads obtained were then subjected to quality control using the fastqc software (http://www.bioinformatics.babraham.ac.uk/projects/fastqc/). For mapping and expression analysis, a reference backbone of 46,898 genes data set, established for MED‐Q1, was used (provided by Prof. Xiao‐Wei Wang, Zhejiang University, China). The data set was manually curated to include the consensus detoxification gene data set described above. The reads were mapped using RSEM (reads per kilobase of transcript per million mapped reads) v1.2.18. The transcript reference was first prepared (rsem‐prepare‐reference), followed by rsem‐calculate‐expression with the parameter Bowtie2. The percentage of mapped reads ranged from 41 to 78.

Genes that did not have at least 10 reads in 4% of the samples were filtered out. The RSEM gene quantification for all the remaining genes was used as input for the deseq2 r package, version 1.10.1. The gene counts were normalized using deseq2 defaults, taking into account the different read mapping percentage of the different samples. Differential expression analysis was performed using a full two‐factorial model. Pairwise comparisons were performed between plants within species and between species between plants. All pairwise comparisons were applied with the parameter “cooksCutoff = FALSE.” False discovery rate (FDR) was corrected for all the 30,012 genes that were not filtered out. The 95% log_2_‐converted fold‐change range (2.5%–97.5% quantiles) was −3.65 to 4.14 between species and −1 to 1.2 within species. Log_2_‐converted fold‐change differences and corrected *p*‐values were considered at 1 and 0.05, respectively, for the constitutive expression comparisons (between species feeding on eggplant) and 0.58 (1.5‐fold expression change) and 0.05, respectively, for the plastic comparisons (within species after transfer from eggplant to cassava, kale or pepper).

Two tests were performed to show that DNA sequence differences between the six *B. tabaci* species did not bias our results due to differences in mapping efficiency. We first performed an analysis of sequence similarity, focusing on the set of 298 detoxification genes. We produced one assembled transcriptome for each of the six analysed species, using RNA‐Seq data from all the species’ RNA samples. Next, we used a “blast reciprocal best hit” approach to check the identity of each gene/contig (of the relevant detoxification gene) in the species’ transcriptome to its putative orthologous gene/contig in the manually curated data set (see above). All reported alignments include genes/contigs that had at least 70% of their sequence aligned with a cut‐off of at least 50% identity. As can be seen in Supporting Information Figure S2, the mean identity for all six species was higher than 95%, meaning that the mean number of mismatches in the mapping process of reads of 75‐bp long was up to ≈3 (1.22–3.31), which is less than that allowed by the default option of Bowtie2. In addition, arcsin‐square root transformed proportions of per cent identities showed only low correlations with estimated deseq2 rld values of the detoxification genes among all possible insect species and plant species combinations (Pearson's *r* ≤ 0.31). Rld stands for regularized log transformation of the original count data to a log_2_ scale by fitting a model with a term for each sample.

For visualizations by principal component analysis (PCA), ANOVA and hierarchical clustering, deseq2 rld values were used. The PCA, ANOVA and hierarchical clustering were performed using the partek ^®^ genomics suite ^®^ software, version 6.6 (v6.6; St. Louis, MO, 2014). The correlation method was applied to calculate the dispersion matrix of the PCA, and the eigenvectors were normalized. ANOVA was used to calculate the mean sources of variation for all the genes (*B. tabaci* and plant species as main effects and their interaction). For the hierarchical clustering, Pearson's dissimilarity and complete linkage were applied.

### 2.7 Verification of differential expression by real‐time polymerase chain reaction

The expression levels of nine detoxification genes that were differentially or equally expressed by the RNA‐Seq approach were validated using quantitative reverse transcription–PCR (qRT–PCR). Comparisons were made between the SSA1‐SG3 and MEAM1 species, which showed the highest (98.33%) and lowest (95.58%) gene identity to the curated data set. The technical details of the qRT‐PCR analyses appear in Supporting Information Table S3. A perfect match was observed between the RNA‐Seq and qRT‐PCR analyses (all qRT‐PCR results are summarized in Supporting Information Table S4).

## 3 RESULTS

### 3.1 Do all the species of the *B. tabaci* complex share a common host range?

**Figure 1:**
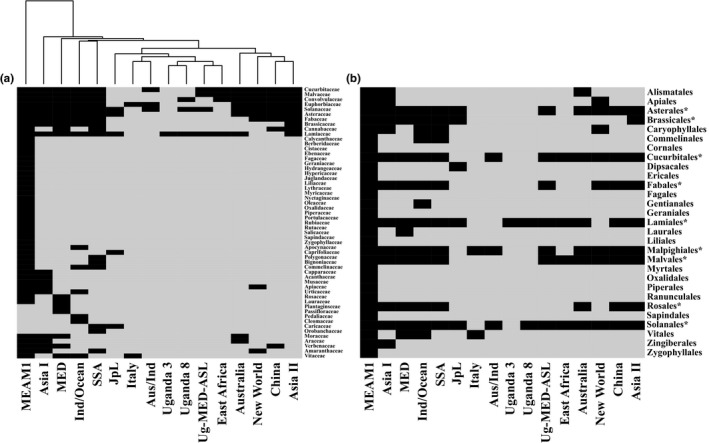
The presumed diet breadth of species groups in the *Bemisia tabaci* complex. Data from Supporting Information Table S1 were used as input for the heatmap.2 function of the r gplots package (R Core Team, 2015). (a) The dendrogram clusters (UPGMA clustering of euclidean distances) the different *B. tabaci* species into four groups according to their botanical families host range. (b) Mapping the data to the host plant orders allows the identification of nine orders (indicated with asterisks) that are commonly shared by most *B. tabaci* species. Names of *B. tabaci* species are displayed according to that of panel (a) for comparative purposes

We first conducted a literature survey in which we documented all of the major sampling efforts of *B. tabaci*. Clustering analysis, at the botanical family level (Figure 1a), indicated that one species in the complex (MEAM1) can be considered as a true “generalist,” four species can be considered to be species with “extended” host ranges (Asia I, Indian Ocean, MED and SSA‐1), while the others can be roughly divided into two groups based on their characteristics of being with a more or less restricted host range. Mapping the data to the host plant orders (Figure 1b) allowed the identification of nine orders (belonging to the Asterids and Rosids clades) that are commonly shared by most *B. tabaci* species: Asterales, Fabales, Rosales, Cucurbitales, Malvales, Malpighiales, Brassicales, Solanales and Lamiales. Reconstruction of ancestral host range (ML algorithm) suggested that only plant species belonging to the Lamiales and Solanales orders can be considered to be ancestral hosts with high probability (*p *≥* *0.91; Supporting Information Figure S1b and Table S5). Further analysis by an MP algorithm showed that the Malpighiales may also be considered as ancestral hosts of *B. tabaci* (Supporting Information Figure S3).

Based on these findings (and species availability), six *B. tabaci* species (representing different geographical and documented host‐range groups) were selected for further analyses: MEAM1, MED‐Q1, Uganda‐MED‐ASL, NW2, Asia II‐1 and SSA1‐SG3. In parallel, four plant species, representing different probabilities of being considered as a common/ancestral host plants, were included in the experimental setting: eggplant, pepper, cassava and kale (more details are provided in section 2.2).

### 3.2 Selected species performance on the various plant hosts

**Figure 2:**
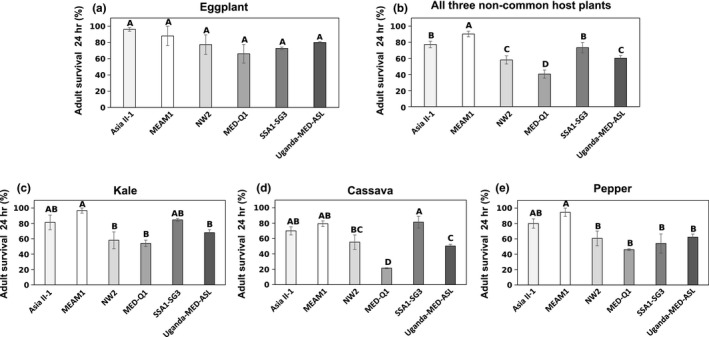
Survival (%) of adults from the six analysed *Bemisia tabaci* species after feeding for 24 hr on the common host plant eggplant (a) or the noncommon host plants: kale, cassava and pepper (b–e). Different letters indicate significant differences (*p* ≤ 0.05, sequential Bonferroni comparisons using the Dunn–Sidak method). Errors bars represent standard error of the means (*N* = 3)

Adult survival was monitored 24 hr after subjecting newly emerged adults from the six selected species to eggplant, cassava, kale and pepper plants. Both main treatments (species and plant host) were found to affect adult survival significantly (*p *<* *0.0001 and *p *=* *0.0001, respectively), but their interaction was not significant (*p *=* *0.148). As expected, the six species did not differ in their survival on eggplant (Figure 2a). Survival over the other three less common hosts (see section 2.2), however, indicated that the six *B. tabaci* species can be divided roughly into two performance groups with MEAM1, Asia II‐1 and SSA1‐SG3, showing higher performance on the various hosts compared to NW2, MED‐Q1 and Uganda‐MED‐ASL (Figure 2b; “high performance” and “low performance” groups, respectively). This result was in general agreement with our literature survey data, which predicted MEAM1 and SSA1‐SG3 to be less specialized compared to NW2 and Uganda‐MED‐ASL. The two exceptions were Asia II‐1, which seemed to be capable of performing well on more hosts than reported in the literature and MED‐Q1, which did not tolerate plant‐by‐plant switches well. Plant‐by‐plant examination indicated that MEAM1 performed significantly better than NW2, MED‐Q1 and Uganda‐MED‐ASL on kale (Figure 2c). SSA1‐SG3 performed significantly better than NW2, MED‐Q1 and Uganda‐MED‐ASL on cassava, while MEAM1 and Asia II‐1 differed significantly only from the latter two (Figure 2d). MEAM1 performed significantly better than NW2, MED‐Q1, SSA1‐SG3 and Uganda‐MED‐ASL on pepper (Figure 2e).

### 3.3 General expression profiles of detoxification genes on the different host plants

**Figure 3:**
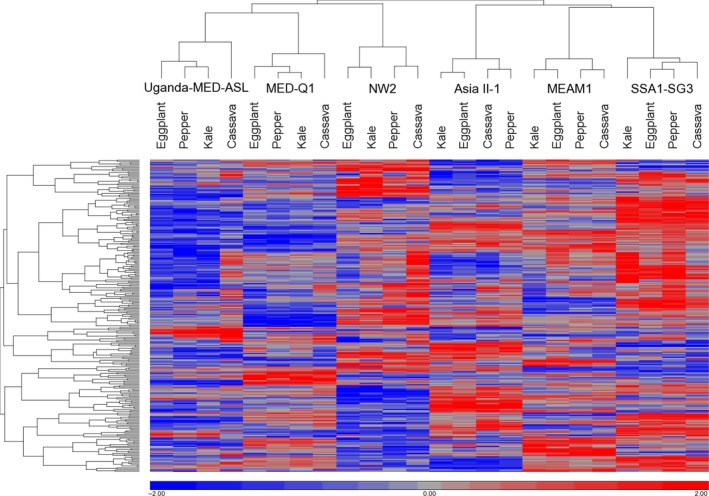
Heat map showing summary of the mean expression patterns of detoxification genes significantly regulated in adults of the six analysed *Bemisia tabaci* species after feeding for 24 hr on the common host plant eggplant or the noncommon host plants: kale, cassava and pepper. Red represents upregulation, and blue represents downregulation (standardization was made on rld values for each gene across all 24 species and plant combinations). Two‐way clustering of genes and samples was performed applying hierarchical clustering using Pearson's dissimilarity for distance measure and complete linkage method for clustering [Colour figure can be viewed at wileyonlinelibrary.com]

We first used ordination and statistical methods to analyse co‐expression between the candidate genes, comparing gene expression values from the six analysed species on the four host plants. PCA showed that the samples group together mainly according to their plant species association (Supporting Information Figure S4). This finding was backed up by two‐way ANOVA on rld values (see section 2.6), which indicated that the majority of variance was associated with differences between species (70.79%). Hierarchical clustering analysis of the differentially expressed genes in at least one comparison (266 genes) was then conducted (Figure 3).

Similar to the PCA and ANOVA findings, the four diet samples of each species clustered together at the first hierarchical level. In addition, there was no clear grouping of samples by host plant within each species. An important result was obtained at the next clustering level (above species), where the detoxification gene expression pattern of the species clustered according to their host‐performance groups (putatively reflecting their more or less specialized state) and not according to their level of phylogenetic relatedness: MEAM1, Asia II‐1 and SSA1‐SG3 in one group, and NW2, MED‐Q1 and Uganda‐MED‐ASL in the other (Figure 3). To verify that this clustering result is meaningful and cannot be repeatedly obtained by random subsampling of the 27,431 differentially expressed genes pool, we ran 500 similar trials of hierarchical clustering analysis using each time a randomly selected set of 266 genes (without replacement). None of these 500 trials clustered the species according to their host‐performance groups.

### 3.4 Constitutive expression differences between *B. tabaci* species

The transcriptomic profile of each species on eggplant (a suitable host plant for all six species, Figure 2a) was used as a baseline, and the constitutive expression differences in detoxification genes were compared. Only genes significantly overexpressed or underexpressed in one species compared to all others were considered.

**Figure 4:**
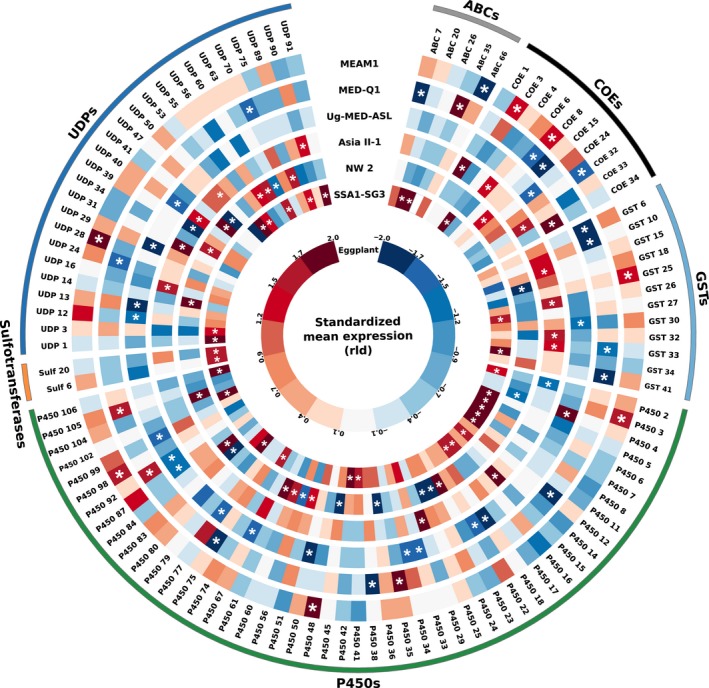
Constitutive expression differences, in detoxification genes, between the six analysed *Bemisia tabaci* species after feeding for 24 hr on the common host plant, eggplant. Genes significantly overexpressed or underexpressed in one species compared to all others are indicated by asterisks. Expression values were plotted as standardized rld values for each gene across the six species. For each listed gene, the Bta number or accession number in the *B. tabaci* MEAM1 genome database (Chen et al., 2016) is provided in Supporting Information Table S2. The circular plot was made with circos [Colour figure can be viewed at wileyonlinelibrary.com]

Overall, from the 298 genes analysed, 105 were significantly constitutively overexpressed or underexpressed in one species (compared to all others), with a slightly higher percentage of overexpressed ones (62%; Figure 4). SSA1‐SG3 showed the highest number of genes that are constitutively overexpressed (29), followed by Asia II‐1 (14) and NW2 (13). On the other hand, MEAM1, MED‐Q1 and Uganda‐MED‐ASL had the lowest number of constitutively overexpressed genes (7, 4 and 2, respectively). A different pattern appeared when genes significantly underexpressed in one species (compared to all others) were considered. Here, there was a remarkable difference between the three species in the “low performance” group, NW2, MED‐Q1 and Uganda‐MED‐ASL, with 10, 12 and 17, underexpressed genes, respectively, to the three “high performance” species, MEAM1, Asia II‐1 and SSA1‐SG3, with 2, 2 and 0, underexpressed genes, respectively.

### 3.5 Plastic expression differences within and between *B. tabaci* species

Comparisons focused on plastic responses (within each species), after a transfer from eggplant to cassava, pepper and kale. Expression data indicated that 151 of the 298 (51%) detoxification genes analysed were plastically expressed. Of these, 95 were plastically expressed in only one species, while 56 were plastically expressed in more than one species. Interestingly, while the relative proportion of the six detoxification gene families in the 95 list did not differ from that of the complete list of the 298 detoxification genes analysed ($\chi_{\left(5\right)}^{2}$ *=* 8.95*, p *=* *0.11), a significant enrichment of genes belonging to the P450 and UDPGT families was observed in the 56 genes’ list ($\chi_{\left(5\right)}^{2}$ *=* 14.52*, p *=* *0.012).

**Figure 5:**
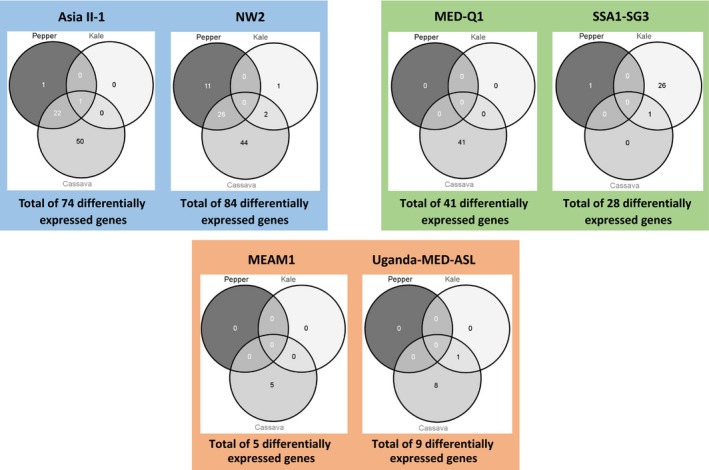
Venn diagrams of differentially regulated detoxification genes within each of the six analysed *Bemisia tabaci* species after switching from the common host plant, eggplant, to the noncommon host plants: kale, cassava and pepper (plastic responses within each species). Log_2_‐converted fold‐change differences and corrected *p*‐values (30,012 analysed genes) were considered at 0.58 (1.5‐fold expression change) and 0.05, respectively. The three main expression patterns, (a) high plastic responses to more than one plant host, (b) high plastic responses to just one plant host, and (c) low level of plastic responses to host transfer, are highlighted in blue, green and orange backgrounds, respectively [Colour figure can be viewed at wileyonlinelibrary.com]

The six species largely differed in the number of detoxification genes showing plastic responses to host transfer (Figure 5), and three main expression patterns were revealed: (a) High plastic response to more than one plant host was seen in Asia II‐1 and NW2 (74 and 84 genes, respectively), which modified their detoxification expression profile largely when transferred to cassava and to a lesser extent to pepper. The majority of genes responding to the transfer to pepper responded also to the transfer to cassava. (b) High plastic response to just one plant host by the MED‐Q1 and SSA1‐SG3 species (41 and 27 genes, respectively), which modified their detoxification expression profile when transferred from eggplant to cassava or kale, respectively. (c) Low level of plastic response to host transfer by MEAM1 and Uganda‐MED‐ASL after transfer from eggplant to cassava (five and nine genes, respectively).

### 3.6 Do *B. tabaci* species share a common “essential detoxification machinery”?

It has been predicted that more specialized species retain an essential “machinery” that allows some level of utilization of host plants that have been lost from the species repertoire (Nylin & Janz, 2009). We asked, therefore, whether a common response in detoxification gene expression exists in *B. tabaci*. To avoid considering nonconsistent changes from which a clear pattern cannot be obtained, only genes differentially expressed in more than one species and showing the same expression pattern (upregulation or downregulation) in at least two species were considered. This reduced the original list of 56 genes, differentially expressed in more than one species, to 44.

**Figure 6:**
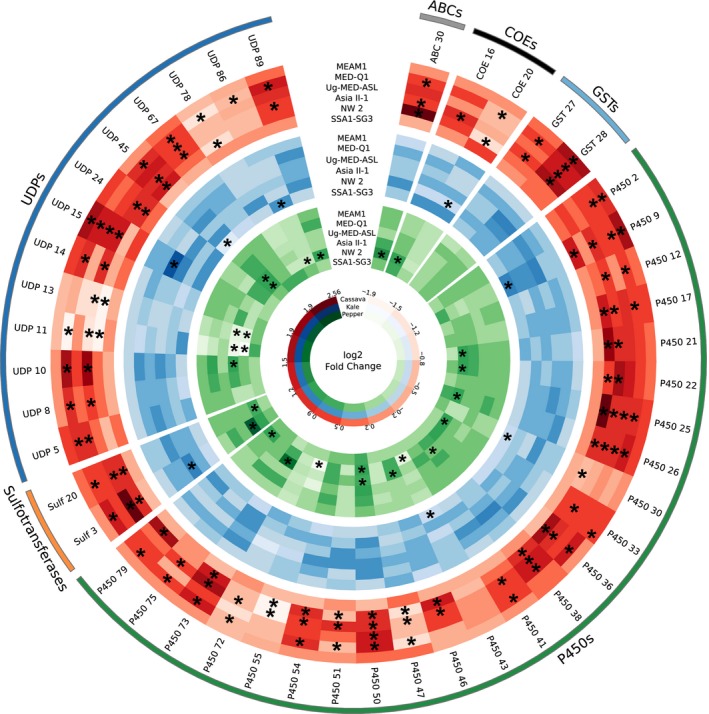
The putative common “essential detoxification machinery” of the *Bemisia tabaci* species complex. The ideogram presents transcripts that were significantly plastically‐expressed (indicated by asterisks) in more than one species and showed the same expression pattern (upregulation or downregulation) in at least two species. For each listed gene, the Bta number or accession number in the *B. tabaci* MEAM1 genome database (Chen et al., 2016) is provided in Supporting Information Table S2. The red, blue and green colours indicate host transfer, to cassava, kale and pepper, respectively. The circular plot was made with circos [Colour figure can be viewed at wileyonlinelibrary.com]

The list of 44 genes (Figure 6) was also significantly enriched in genes belonging to the P450 (24 genes) and UDPGT (13 genes) detoxification families ($\chi_{\left(5\right)}^{2}$ *=* 14.85*, p *=* *0.011). Six genes were upregulated in response to host switch in four species; 12 were upregulated and two were downregulated in three species; and 15 were upregulated and nine were downregulated in two species. Two interesting observations were made: First, in nearly all genes (41 of 44), the plastic changes in expression in all species and on all host plants occurred in the same direction (upregulation or downregulation). Second, with five exceptions (out of 29 cases), the detoxification genes, which plastically responded to host transfer from eggplant to pepper and kale, were the same ones that responded to the transfer to cassava, possibly suggesting the existence of a commonly induced detoxification gene set, capable of neutralizing a wide and unrelated range of phytotoxins.

## 4 DISCUSSION

We present here three independent lines of evidence to suggest that species within the *B. tabaci* complex differ both in their ability to accept or utilize multiple plant hosts and in their detoxification expression patterns. First, our literature survey of field collection data clearly indicated that only a few species in the complex can be found on hosts from multiple botanical families, while the majority of species were limited to only few families. Second, our adult survival assays separated the six species into two performance groups, showing higher (MEAM1, Asia II‐1 and SSA1‐SG3) or lower (NW2, MED‐Q1 and Uganda‐MED‐ASL) ability to survive on noncommon and toxic plant hosts, putatively reflecting the possibility that the two groups differ in their ability to utilize multiple hosts under field conditions. Third, hierarchical clustering analysis of their differentially expressed detoxification genes clustered the six species according to their aforementioned host‐performance groups and not according to their phylogenetic relationship, bringing evidence for the existence of a common detoxification “machinery,” shared between species that can perform well on multiple hosts.

The hypothesis that the different species in the *B. tabaci* complex differ in their “actual” host range is definitely not a new one (Brown et al., 1995), although it was rightly argued that this is more a hypothesis than a “solid” fact, as there are only few experimental studies that compared performance across different hosts (De Barro et al., 2011; Xu et al., 2011). What is new here is the possible link we draw between divergence in this group and the documented differences in the species’ host‐plant ranges. The very few studies that previously considered the possibility that host plants played a role in the complex divergence did not provide a clear statement, but argued that this is unlikely because most species in the complex have the ability to utilize multiple hosts (De Barro, 2005; De Barro et al., 2005). Alternatively, it was hypothesized that the host range of each of the various species identified in sympatry is fairly equivalent and that it is possible that, in small spatial scales, density‐dependent competitive interactions have operated to exclude an invader belonging to a different species (De Barro, 2005; De Barro et al., 2011).

Our transcriptomic findings add a new dimension to these previous data. As stated above, the hierarchical clustering analysis clustered the six species according to their ability to perform on multiple hosts. This suggests the existence of a shared detoxification gene expression pattern that can be associated with a more general feeding nature of some species, regardless of the estimated time of their separation. Moreover, it allows us to speculate that these species have likely retained an ancestral pattern of expression that was already present in the common ancestor of the *B. tabaci* species complex, which likely displayed a feeding habit towards the less specialized side of the *B. tabaci* spectrum. It is important to note that our data do not allow us to exclude the possibility that convergent evolution to similar detoxification patterns has taken place (Ujvari et al., 2015) in species displaying a more general feeding nature.

We would like to turn back now to our main research question: “Could evolved differences in host utilization play a role in the diversification of the *B. tabaci* species complex?” Our literature survey data clearly indicated that the *B. tabaci* species complex should be recognized as a group of more or less specialized species. In parallel, we found evidence for the maintenance of a common (ancestral or converged) expression pattern of the detoxification “machinery” that is shared among species that can perform well on multiple common and novel hosts. As species with expanded host ranges tend to show larger geographical distributions, they are more susceptible to fragmentation due to both neutral and adaptive processes (Forister & Jenkins, 2017; Forister et al., 2012; Janz, Braga, Wahlberg, & Nylin, 2016; Nyman, 2010). Therefore, it is possible that the ancestral ability to perform well on multiple hosts might have played a passive role in the evolution of the *B. tabaci* species complex, by enhancing the probability for geographical separation between populations. Under this scenario, most of the subsequent divergence, including some adaptation/specialization events, could have occurred in allopatry, fitting the observations and predictions made by De Barro (2005).

Few additional interesting and likely important observations were made while exploring our transcriptomic data. For example, some common detoxification capabilities were found to be lost only by species that cannot perform well on multiple hosts, although it seems that these species did not converge to one “other” pattern. This suggests that genetic drift or selection pressure might cause the loss of some detoxification genes that are not required when species become more specialized and lose their ability to perform well on some host plants. It has been previously argued that production of detoxification proteins might be energetically costly or capable of endangering the organism by producing modified/bio‐activated deleterious molecules (Feyereisen, 1999). For example, β‐asarone bio‐activation was mediated via insect P450 activity in *Peridroma saueiaas* (Koul, Smirle, Isman, & Szeto, 1990) and P450 activity in *Helicoverpa zea*, was detrimental in the presence of a plant pathogen that produces aflatoxin, a toxin that can be bio‐activated by P450s activity (Zeng, Wen, Niu, Schuler, & Berenbaum, 2009). Other examples include mitochondrion‐associated transcripts or chaperonin responses that can mitigate the effect of stressful or foreign environments but are also associated with major energetic costs (Feder & Hofmann, 1999; Ragland et al., 2015).

Our transcriptomic data also provide new insights into the mode of expression of detoxification genes in generalist phloem feeders. It has been argued that detoxification genes in generalist species should show high level of plasticity upon host switches due to their crucial role in early mitigation of the new host defensive chemistry (Celorio‐Mancera et al., 2013; Grbić et al., 2011; Vogel, Musser, & Celorio‐Mancera, 2014; Wybouw et al., 2015). At the same time, these genes could be primary targets of natural selection (Celorio‐Mancera et al., 2013; Roy et al., 2016) that may fix, via genetic accommodation, adaptive changes that decrease the environmental responsiveness of the genes (Schlichting & Wund, 2014), leading to their environmental‐independent (constitutive) expression.

Unfortunately, our study does not provide conclusive evidence to support any of these predictions. Still, it can be carefully stated that, in the *B. tabaci* species complex, plasticity in detoxification gene expression seems not to be associated with the ability to perform well on multiple hosts. Moreover, the ability to constitutively overexpress detoxification genes was also only partially correlated with better ability to perform well on novel hosts. While two of the less specialized species, SSA1‐SG3 and Asia II‐1, showed the highest number of genes that are constitutively overexpressed (29 and 14, respectively), MEAM1, the most generalist species in the *B. tabaci* complex, uniquely overexpressed only seven genes.

We should emphasize here that large‐scale gene expression analyses clarifying the molecular mechanisms of host alternation and adaptation in other major generalist phloem‐feeding species are extremely limited. A recent study on the generalist aphid species, *M. persicae*, compared colonies that were reared in parallel for 1 year on *Brassica rapa* or *Nicotiana benthamiana*. Comparison of the colony's transcriptomes identified only 171 differentially expressed genes putatively involved in host adjustment from a total of >18,000 genes (<1%). Moreover, the *B. rapa* clone successfully colonized *N. benthamiana* with no significant differences observed in its performance (Mathers et al., 2017). Although the outlined experimental system differed from ours, the common findings in the two systems highlight the possibility that successful short‐ or long‐term host shifts of generalist phloem feeders do not necessarily require significant plastic or constitutive changes in gene expression. Another striking similarity between our study and the one described by Mathers et al. (2017) relates to the enrichment, in both systems, of differentially expressed genes from to the P450 and UDPGT families responding to host changes. Interestingly, Mathers et al. (2017) noticed that these genes have a tendency to be arranged as tandem repeats in the *M. persicae* genome, drawing a putative link between gene duplication/family expansion events, expression plasticity and macro‐evolutionary processes involved in host adaptation (Edger et al., 2015).

Before concluding, we would like to highlight few important noncompleted matters that require further investigation. First, although our transcriptomic study is the most extensive one conducted so far on the *B. tabaci* complex, it is clear that analyses of more combinations of species/plant hosts are required for further solidification of our findings and insights. Second, our literature survey included multiple sampling attempts from every relevant continent spanning more than 20 years. Therefore, we consider the effect of sampling bias negligible. Still, for some specific species, especially in the Asia II and China genetic groups, more data are required in order to determine the species “true” host range. In addition, it is also possible that some species in the complex, identified in the literature survey as having an “extended” host range, are in fact a mixture of individual genotypes that are adapted to certain plant types (Loxdale & Harvey, 2016). This did not affect our study, as each species was represented by one genotype (specific mtCOI barcode), but should be taken into consideration when comparisons to other studies are made in the future. Third, due to the lack of reliable annotated data, our work (so far) focused only on the detoxification system. Other systems that insects require for feeding successfully on their host plants should be targeted in the future. These include among others: systems that allow host‐plant perception and continued feeding through olfactory and/or gustatory cues, digestion and plant nutrient uptake systems, and the management of parasites present in the insect diet (Koenig et al., 2015). Fourth, it should be noted that “gene expression” could be also regulated at the translational and post‐translational levels. Detoxification genes that were identified as being expressed differentially across species (“constitutive” genes) and those with induced or suppressed RNA levels after host shift (“plastic” genes) may have an added layer of regulatory complexity that were not revealed in this study and should be addressed in the future. For instance, amino acid residue polymorphisms across the six *B. tabaci* species or possible changes in the levels of the detoxification proteins (due to altered translation or turnover).

We would like to conclude by highlighting few of our findings that might apply across other phytophagous insect systems. First, common detoxification “machineries” that allow the successful utilization of multiple plant hosts and the exploration of new/novel environments are likely to exist in other generalist species complexes. Second, our findings provide an insight into how more generalized and more specialized genotypes evolve. They raise the possibility that successful short‐ or long‐term host shifts of generalist phloem feeders do not necessarily require significant plastic or constitutive changes in gene expression, which makes this feeding guild quite different from other studied systems (Celorio‐Mancera et al., 2013; Ragland et al., 2015; Wybouw et al., 2015). Third, they also provide a mechanistic platform for explaining why specialization should not be considered as a dead end, as even more specialized species retained parts of the environmental‐responsive detoxification “machinery.” We hypothesize that this essential “machinery” is in general adaptive, as selection has had an opportunity to act on the genetic variation for plasticity throughout most of the host range of the species. It might even allow populations of more specialized species to survive to some extent on novel hosts (Nylin & Janz, 2009). It is important to note, however, that the way forward, especially in nonmodel organisms, requires the integration of high‐quality genomic and epigenomic data, which will allow to accurately define the interplay between genotypes and phenotypes and between ecological and evolutionary processes (Forister et al., 2012; Schneider & Meyer, 2017).

## DATA ACCESSIBILITY

Raw data files from the RNA sequencing project described above have been deposited in the ncbi Short Read Archive SRP127757.

## AUTHOR CONTRIBUTION

Experimental design and execution, RNA extraction, submission of samples for sequencing, data analysis and writing were carried out by O.M. Contribution to processing of raw sequencing data, transcriptomes assembly, comparative transcriptomic analysis, phylogenetics, ancestral host‐range reconstruction analyses, writing and production of figures was made by D.S.G. Processing of raw sequencing data, gene expression analysis, writing and production of figures were carried out by E.F. qRT‐PCR validation and writing were carried out by E.S. Sample collection and writing were done by R.K.S., H.D. and H.M. Experimental execution, RNA extraction and writing were carried out by S.V.B. Gene annotation was performed by M.P. and P.V. Study conception, experimental design, RNA extraction and writing were carried out by S.S. Study conception, experimental design and execution, sample collection and writing were done by J.C. Literature survey, study conception and experimental design, project management, data analysis and writing were carried out by S.M.

## References

[mec14865-bib-0001] Alves, A. A. C. (2002). Cassava botany and physiology In Hillocks R. J., Thresh J. M., & Bellotti A. C. (Eds.), Cassava biology, production and utilization (pp. 67–89). New York, NY: CABI Publishing 10.1079/9780851995243.0000

[mec14865-bib-0002] Antwi, J. B. , Sword, G. A. , & Medina, R. F. (2015). Host‐associated differentiation in a highly polyphagous, sexually reproducing insect herbivore. Ecology and Evolution, 5, 2533–2543. 10.1002/ece3.1526 26257868 PMC4523351

[mec14865-bib-0003] Barbosa, L. F. , Marubayashi, J. M. , De Marchi, B. R. , Yuki, V. A. , Pavan, M. A. , Moriones, E. , … Krause‐Sakatea, R. (2014). Indigenous American species of the *Bemisia tabaci* complex are still widespread in the Americas. Pest Management Science, 70, 1440–1445. 10.1002/ps.3731 24458534

[mec14865-bib-0004] Berlocher, S. H. , & Feder, J. L. (2002). Sympatric speciation in phytophagous insects: Moving beyond controversy? Annual Review of Entomology, 47, 773–815. 10.1146/annurev.ento.47.091201.145312 11729091

[mec14865-bib-0005] Bickford, D. , Lohman, D. J. , Sodhi, N. S. , Ng, P. K. , Meier, R. , Winker, K. , … Das, I. (2007). Cryptic species as a window on diversity and conservation. Trends in Ecology & Evolution, 22, 148–155. 10.1016/j.tree.2006.11.004 17129636

[mec14865-bib-0006] Boykin, L. M. , Bell, C. D. , Evans, G. , Small, I. , & De Barro, P. J. (2013). Is agriculture driving the diversification of the *Bemisia tabaci* species complex (Hemiptera: Sternorrhyncha: Aleyrodidae)?: Dating, diversification and biogeographic evidence revealed. BMC Evolutionary Biology, 13, 228 10.1186/1471-2148-13-228 24138220 PMC3853546

[mec14865-bib-0007] Brown, J. K. , Frohlich, D. R. , & Rosell, R. C. (1995). The sweetpotato or silverleaf whiteflies: Biotypes of *Bemisia tabaci* or a species complex? Annual Review of Entomology, 40, 511–534. 10.1146/annurev.en.40.010195.002455

[mec14865-bib-0008] Celorio‐Mancera, M. D. , Wheat, C. W. , Vogel, H. , Söderlind, L. , Janz, N. , & Nylin, S. (2013). Mechanisms of macroevolution: Polyphagous plasticity in butterfly larvae revealed by RNA‐Seq. Molecular Ecology, 22, 4884–4895. 10.1111/mec.12440 23952264

[mec14865-bib-0009] Chen, W. , Hasegawa, D. K. , Kaur, N. , Kliot, A. , Pinheiro, P. V. , Luan, J. , … Xu, Y. (2016). The draft genome of whitefly *Bemisia tabaci* MEAM1, a global crop pest, provides novel insights into virus transmission, host adaptation, and insecticide resistance. BMC Biology, 14, 110 10.1186/s12915-016-0321-y 27974049 PMC5157087

[mec14865-bib-0010] Colvin, J. , Omongo, C. A. , Maruthi, M. N. , Otim‐Nape, G. W. , & Thresh, J. M. (2004). Dual begomovirus infections and high *Bemisia tabaci* populations: Two factors driving the spread of a cassava mosaic disease pandemic. Plant Pathology, 53, 577–584. 10.1111/j.0032-0862.2004.01062.x

[mec14865-bib-0011] Cornell, H. V. , & Hawkins, B. A. (2003). Herbivore responses to plant secondary compounds: A test of phytochemical coevolution theory. The American Naturalist, 161, 507–522. 10.1086/368346 12776881

[mec14865-bib-0012] De Barro, P. J. (2005). Genetic structure of the whitefly *Bemisia tabaci* in the Asia‐Pacific region revealed using microsatellite markers. Molecular Ecology, 14, 3695–3718. 10.1111/j.1365-294X.2005.02700.x 16202090

[mec14865-bib-0013] De Barro, P. , & Bourne, A. (2010). Ovipositional host choice by an invader accelerates displacement of its indigenous competitor. Biological Invasions, 12, 3013–3023. 10.1007/s10530-010-9691-1

[mec14865-bib-0014] De Barro, P. J. , Liu, S. S. , Boykin, L. M. , & Dinsdale, A. B. (2011). *Bemisia tabaci*: A statement of species status. Annual Review of Entomology, 56, 1–19. 10.1146/annurev-ento-112408-085504 20690829

[mec14865-bib-0015] De Barro, P. J. , Trueman, J. W. H. , & Frohlich, D. R. (2005). *Bemisia argentifolii* is a race of *B. tabaci* (Hemiptera: Aleyrodidae): The molecular genetic differentiation of *B. tabaci* populations around the world. Bulletin of Entomological Research, 95, 193–203. 10.1079/BER2004351 15960874

[mec14865-bib-0016] Dermauw, W. , Pym, A. , Bass, C. , Van Leeuwen, T. , & Feyereisen, R. (2018). Does host plant adaptation lead to pesticide resistance in generalist herbivores? Current Opinion in Insect Science, 26, 25–33. 10.1016/j.cois.2018.01.001 29764657

[mec14865-bib-0017] Dermauw, W. , Wybouw, N. , Rombauts, S. , Menten, B. , Vontas, J. , Grbić, M. , … Van Leeuwen, T. (2013). A link between host plant adaptation and pesticide resistance in the polyphagous spider mite *Tetranychus urticae* . Proceedings of the National Academy of Sciences of the United States of America, 110, E113–E122. 10.1073/pnas.1213214110 23248300 PMC3545796

[mec14865-bib-0018] Després, L. , David, J. P. , & Gallet, C. (2007). The evolutionary ecology of insect resistance to plant chemicals. Trends in Ecology & Evolution, 22, 298–307. 10.1016/j.tree.2007.02.010 17324485

[mec14865-bib-0019] Dinsdale, A. , Cook, L. , Riginos, C. , Buckley, Y. M. , & De Barro, P. (2010). Refined global analysis of *Bemisia tabaci* (Hemiptera: Sternorrhyncha: Aleyrodoidea: Aleyrodidae) mitochondrial cytochrome oxidase 1 to identify species level genetic boundaries. Annals of the Entomological Society of America, 103, 196–208. 10.1603/AN09061

[mec14865-bib-0020] Edger, P. P. , Heidel‐Fischer, H. M. , Bekaert, M. , Rota, J. , Glöckner, G. , Platts, A. E. , … Wheat, C. W. (2015). The butterfly plant arms‐race escalated by gene and genome duplications. Proceedings of the National Academy of Sciences, 112, 8362–8366. 10.1073/pnas.1503926112 PMC4500235 26100883

[mec14865-bib-0021] Ellango, R. , Singh, S. T. , Rana, V. S. , Gayatri Priya, N. , Raina, H. , Chaubey, R. , … Rajagopal, R. (2015). Distribution of *Bemisia tabaci* genetic groups in India. Environmental Entomology, 44, 1258–1264. 10.1093/ee/nvv062 26314072

[mec14865-bib-0022] Feder, M. E. , & Hofmann, G. E. (1999). Heat‐shock proteins, molecular chaperones, and the stress response: Evolutionary and ecological physiology. Annual Review of Physiology, 61, 243–282. 10.1146/annurev.physiol.61.1.243 10099689

[mec14865-bib-0023] Feyereisen, R. (1999). Insect P450 enzymes. Annual Review of Entomology, 44, 507–533. 10.1146/annurev.ento.44.1.507 9990722

[mec14865-bib-0024] Forister, M. L. , Dyer, L. A. , Singer, M. S. , Stireman, J. O. , & Lill, J. T. (2012). Revisiting the evolution of ecological specialization, with emphasis on insect–plant interactions. Ecology, 93, 981–991. 10.1890/11-0650.1 22764485

[mec14865-bib-0025] Forister, M. L. , & Jenkins, S. H. (2017). A neutral model for the evolution of diet breadth. The American Naturalist, 190, E40–E54. 10.1086/692325 28731794

[mec14865-bib-0026] Forister, M. L. , Novotny, V. , Panorska, A. K. , Baje, L. , Basset, Y. , Butterill, P. T. , … Drozd, P. (2015). The global distribution of diet breadth in insect herbivores. Proceedings of the National Academy of Sciences of the United States of America, 112, 442–447. 10.1073/pnas.1423042112 25548168 PMC4299246

[mec14865-bib-0027] Fu, L. , Niu, B. , Zhu, Z. , Wu, S. , & Li, W. (2012). CD‐HIT: Accelerated for clustering the next‐generation sequencing data. Bioinformatics, 28, 3150–3152. 10.1093/bioinformatics/bts565 23060610 PMC3516142

[mec14865-bib-0028] Grbić, M. , Van Leeuwen, T. , Clark, R. M. , Rombauts, S. , Rouzé, P. , Grbić, V. , … Hernández‐Crespo, P. (2011). The genome of *Tetranychus urticae* reveals herbivorous pest adaptations. Nature, 479, 487–492. 10.1038/nature10640 22113690 PMC4856440

[mec14865-bib-0029] Heckel, D. G. (2014). Insect detoxification and sequestration strategies In Voelckel C., & Jander G. (Eds.), Annual plant reviews volume 47: Insect‐plant interactions (pp. 77–114). Oxford, UK: Wiley 10.1002/9781118829783

[mec14865-bib-0030] Hereward, J. P. , Walter, G. H. , DeBarro, P. J. , Lowe, A. J. , & Riginos, C. (2013). Gene flow in the green mirid, *Creontiades dilutus* (Hemiptera: Miridae), across arid and agricultural environments with different host plant species. Ecology and Evolution, 3, 807–821. 10.1002/ece3.510 23610626 PMC3631396

[mec14865-bib-0031] Hu, J. , Jiang, Z. L. , Nardi, F. , Liu, Y. Y. , Luo, X. R. , Li, H. X. , & Zhang, Z. K. (2014). Members of *Bemisia tabaci* (Hemiptera: Aleyrodidae) Cryptic Species and the Status of Two Invasive Alien Species in the Yunnan Province (China). Journal of Insect Science, 14, 281. 25502045 10.1093/jisesa/ieu143 PMC5657883

[mec14865-bib-0032] Iida, H. , Kitamura, T. , & Honda, K. I. (2009). Comparison of egg‐hatching rate, survival rate and development time of the immature stage between B‐and Q‐biotypes of *Bemisia tabaci* (Gennadius) (Homoptera: Aleyrodidae) on various agricultural crops. Applied Entomology and Zoology, 44, 267–273. 10.1303/aez.2009.267

[mec14865-bib-0033] Janz, N. , Braga, M. P. , Wahlberg, N. , & Nylin, S. (2016). On oscillations and flutterings—A reply to Hamm and Fordyce. Evolution, 7, 1150–1155. 10.1111/evo.12927 27094253

[mec14865-bib-0034] Janz, N. , & Nylin, S. Ö. R. E. N. (2008). The oscillation hypothesis of host‐plant range and speciation In Tilmon K. J. (Ed.), Specialization, speciation, and radiation: The evolutionary biology of herbivorous insects (pp. 203–215). Berkeley, CA: University of California Press.

[mec14865-bib-0035] Koenig, C. , Bretschneider, A. , Heckel, D. G. , Grosse‐Wilde, E. , Hansson, B. S. , & Vogel, H. (2015). The plastic response of *Manduca sexta* to host and non‐host plants. Insect Biochemistry and Molecular Biology, 63, 72–85. 10.1016/j.ibmb.2015.06.001 26070471

[mec14865-bib-0036] Koul, O. , Smirle, M. J. , Isman, M. B. , & Szeto, Y. S. (1990). Synergism of a natural insect growth inhibitor is mediated by bioactivation. Experientia, 46, 1082–1084. 10.1007/BF01940681

[mec14865-bib-0037] Lazarević, J. , Janković‐Tomanić, M. , Savković, U. , Đorđević, M. , Milanović, S. , & Stojković, B. (2017). Host‐associated divergence in the activity of digestive enzymes in two populations of the gypsy moth *Lymantria dispar* (Lepidoptera: Erebidae). Entomological Science, 20, 189–194. 10.1111/ens.12250

[mec14865-bib-0038] Lee, W. , Park, J. , Lee, G. S. , Lee, S. , & Akimoto, S. I. (2013). Taxonomic status of the *Bemisia tabaci* complex (Hemiptera: Aleyrodidae) and reassessment of the number of its constituent species. PLoS One, 8, e63817 10.1371/journal.pone.0063817 23675507 PMC3652838

[mec14865-bib-0039] Levis, N. A. , & Pfennig, D. W. (2016). Evaluating ‘plasticity‐first’ evolution in nature: Key criteria and empirical approaches. Trends in Ecology & Evolution, 31, 563–574. 10.1016/j.tree.2016.03.012 27067134

[mec14865-bib-0040] Liu, S. S. , Colvin, J. , & De Barro, P. J. (2012). Species concepts as applied to the whitefly *Bemisia tabaci* systematics: How many species are there? Journal of Integrative Agriculture, 11, 176–186. 10.1016/S2095-3119(12)60002-1

[mec14865-bib-0042] Loxdale, H. D. , & Harvey, J. A. (2016). The ‘generalism’ debate: Misinterpreting the term in the empirical literature focusing on dietary breadth in insects. Biological Journal of the Linnean Society, 119, 265–282. 10.1111/bij.12816

[mec14865-bib-0043] Margaritopoulos, J. T. , Malarky, G. , Tsitsipis, J. A. , & Blackman, R. L. (2007). Microsatellite DNA and behavioural studies provide evidence of host‐mediated speciation in *Myzus persicae* (Hemiptera: Aphididae). Biological Journal of the Linnean Society, 91, 687–702. 10.1111/j.1095-8312.2007.00828.x

[mec14865-bib-0044] Marques, J. F. , Wang, H. L. , Svensson, G. P. , Frago, E. , & Anderbrant, O. (2014). Genetic divergence and evidence for sympatric host‐races in the highly polyphagous brown tail moth, *Euproctis chrysorrhoea* (Lepidoptera: Erebidae). Evolutionary Ecology, 28, 829–848. 10.1007/s10682-014-9701-3

[mec14865-bib-0045] Mathers, T. C. , Chen, Y. , Kaithakottil, G. , Legeai, F. , Mugford, S. T. , Baa‐Puyoulet, P. , … Dalmay, T. (2017). Rapid transcriptional plasticity of duplicated gene clusters enables a clonally reproducing aphid to colonise diverse plant species. Genome Biology, 18, 27 10.1186/s13059-016-1145-3 28190401 PMC5304397

[mec14865-bib-0046] Matzkin, L. M. (2012). Population transcriptomics of cactus host shifts in *Drosophila mojavensis* . Molecular Ecology, 21, 2428–2439. 10.1111/j.1365-294X.2012.05549.x 22512269

[mec14865-bib-0047] Mokhtar, M. , Soukup, J. , Donato, P. , Cacciola, F. , Dugo, P. , Riazi, A. , … Mondello, L. (2015). Determination of the polyphenolic content of a *Capsicum annuum* L. extract by liquid chromatography coupled to photodiode array and mass spectrometry detection and evaluation of its biological activity. Journal of Separation Science, 38, 171–178. 10.1002/jssc.201400993 25378270

[mec14865-bib-0048] Mugerwa, H. , Seal, S. , Wang, H. L. , Patel, M. V. , Kabaalu, R. , Omongo, C. A. , & Colvin, J. (2018). African ancestry of New World, *Bemisia tabaci*‐whitefly species. Scientific Reports, 8, 2734 10.1038/s41598-018-20956-3 29426821 PMC5807539

[mec14865-bib-0049] Nguyen, L. T. , Schmidt, H. A. , von Haeseler, A. , & Minh, B. Q. (2014). IQ‐TREE: A fast and effective stochastic algorithm for estimating maximum‐likelihood phylogenies. Molecular Biology and Evolution, 32, 268–274. 25371430 10.1093/molbev/msu300 PMC4271533

[mec14865-bib-0050] Nylin, S. , & Janz, N. (2009). Butterfly host plant range: An example of plasticity as a promoter of speciation*?* Evolutionary Ecology, 23, 137–146. 10.1007/s10682-007-9205-5

[mec14865-bib-0051] Nyman, T. (2010). To speciate, or not to speciate? Resource heterogeneity, the subjectivity of similarity, and the macroevolutionary consequences of niche‐width shifts in plant‐feeding insects. Biological Reviews, 85, 393–411. 10.1111/j.1469-185X.2009.00109.x 20002390

[mec14865-bib-0052] Paradis, E. , Strimmer, K. , Claude, J. , Jobb, G. , Opgen‐Rhein, R. , Dutheil, J. , … Lemon, J. (2004). ape: Analyses of phylogenetics and evolution. R package version 1.3‐1. Vienna, Austria: Comprehensive R Archive Network.

[mec14865-bib-0053] Peccoud, J. , Simon, J. C. , von Dohlen, C. , Coeur d'acier, A. , Plantegenest, M. , Vanlerberghe‐Masutti, F. , & Jousselin, E. (2010). Evolutionary history of aphid‐plant associations and their role in aphid diversification. Comptes Rendus Biologies, 333, 474–487. 10.1016/j.crvi.2010.03.004 20541159

[mec14865-bib-0054] Perring, T. M. (2001). The *Bemisia tabaci* species complex. Crop Protection, 20, 725–737. 10.1016/S0261-2194(01)00109-0

[mec14865-bib-0055] Pfennig, D. W. , & Ehrenreich, I. M. (2014). Towards a gene regulatory network perspective on phenotypic plasticity, genetic accommodation and genetic assimilation. Molecular Ecology, 23, 4438–4440. 10.1111/mec.12887 25208504 PMC4180264

[mec14865-bib-0056] Prawat, H. , Mahidol, C. , Ruchirawat, S. , Prawat, U. , Tuntiwachwuttikul, P. , Tooptakong, U. , … White, A. H. (1995). Cyanogenic and non‐cyanogenic glycosides from *Manihot esculenta* . Phytochemistry, 40, 1167–1173. 10.1016/0031-9422(95)00398-Q 7492370

[mec14865-bib-0057] Qin, L. , Pan, L. L. , & Liu, S. S. (2016). Further insight into reproductive incompatibility between putative cryptic species of the *Bemisia tabaci* whitefly complex. Insect Science, 23, 215–224. 10.1111/1744-7917.12296 27001484

[mec14865-bib-0500] R Core Team (2015). R: A language and environment for statistical computing. Vienna, Austria: R Foundation for Statistical Computing.

[mec14865-bib-0501] R Core Team (2017). R: A language and environment for statistical computing. Vienna, Austria: R Foundation for Statistical Computing.

[mec14865-bib-0058] Ragland, G. J. , Almskaar, K. , Vertacnik, K. L. , Gough, H. M. , Feder, J. L. , Hahn, D. A. , & Schwarz, D. (2015). Differences in performance and transcriptome‐wide gene expression associated with *Rhagoletis* (Diptera: Tephritidae) larvae feeding in alternate host fruit environments. Molecular Ecology, 24, 2759–2776. 10.1111/mec.13191 25851077

[mec14865-bib-0059] Roy, A. , Walker, W. B. , Vogel, H. , Chattington, S. , Larsson, M. C. , Anderson, P. , … Schlyter, F. (2016). Diet dependent metabolic responses in three generalist insect herbivores *Spodoptera* spp. Insect Biochemistry and Molecular Biology, 71, 91–105. 10.1016/j.ibmb.2016.02.006 26908076

[mec14865-bib-0060] Rundle, H. D. , & Nosil, P. (2005). Ecological speciation. Ecology Letters, 8, 336–352. 10.1111/j.1461-0248.2004.00715.x

[mec14865-bib-0061] Schlichting, C. D. , & Wund, M. A. (2014). Phenotypic plasticity and epigenetic marking: An assessment of evidence for genetic accommodation. Evolution, 68, 656–672. 10.1111/evo.12348 24410266

[mec14865-bib-0062] Schmidt, S. , Zietz, M. , Schreiner, M. , Rohn, S. , Kroh, L. W. , & Krumbein, A. (2010). Identification of complex, naturally occurring flavonoid glycosides in kale (*Brassica oleracea* var. sabellica) by high‐performance liquid chromatography diode‐array detection/electrospray ionization multi‐stage mass spectrometry. Rapid Communications in Mass Spectrometry, 24, 2009–2022. 10.1002/rcm.4605 20552580

[mec14865-bib-0063] Schneider, R. F. , & Meyer, A. (2017). How plasticity, genetic assimilation and cryptic genetic variation may contribute to adaptive radiations. Molecular Ecology, 26, 330–350. 10.1111/mec.13880 27747962

[mec14865-bib-0064] Simon, J. C. , d'Alençon, E. , Guy, E. , Jacquin‐Joly, E. , Jaquiéry, J. , Nouhaud, P. , … Streiff, R. (2015). Genomics of adaptation to host‐plants in herbivorous insects. Briefings in Functional Genomics, 14, 413–423. 10.1093/bfgp/elv015 25846754

[mec14865-bib-0065] Sokal, R. R. , & Rohlf, F. J. (1995). Biometry (3rd ed.). New York, NY: WH Freeman and Co.

[mec14865-bib-0066] Stireman, J. O. , Nason, J. D. , & Heard, S. B. (2005). Host‐associated genetic differentiation in phytophagous insects: General phenomenon or isolated exceptions? Evidence from a goldenrod‐insect community. Evolution, 59, 2573–2587. 10.1554/05-222.1 16526505

[mec14865-bib-0067] Struck, T. H. , Feder, J. L. , Bendiksby, M. , Birkeland, S. , Cerca, J. , Gusarov, V. I. , … Stedje, B. (2017). Finding evolutionary processes hidden in cryptic species. Trends in Ecology & Evolution, 33, 153–163. 29241941 10.1016/j.tree.2017.11.007

[mec14865-bib-0068] Ujvari, B. , Casewell, N. R. , Sunagar, K. , Arbuckle, K. , Wüster, W. L. N. , O'Meally, D. , … Madsen, T. (2015). Widespread convergence in toxin resistance by predictable molecular evolution. Proceedings of the National Academy of Sciences, 112, 11911–11916. 10.1073/pnas.1511706112 PMC4586833 26372961

[mec14865-bib-0069] Vogel, H. , Musser, R. O. , & Celorio‐Mancera, M. L. (2014). Transcriptome responses in herbivorous insects towards host plant and toxin feeding In Voelckel C. & Jander G. (Eds), Annual plant reviews volume 47: Insect‐plant interactions (pp. 197–233). Oxford, UK: Wiley 10.1002/9781118829783

[mec14865-bib-0070] Wang, H. , Holloway, J. D. , Janz, N. , Braga, M. P. , Wahlberg, N. , Wang, M. , & Nylin, S. (2017). Polyphagy and diversification in tussock moths: Support for the oscillation hypothesis from extreme generalists. Ecology and Evolution, 7, 7975–7986. 10.1002/ece3.3350 29043049 PMC5632610

[mec14865-bib-0071] Wernersson, R. , & Pedersen, A. G. (2003). RevTrans: Multiple alignment of coding DNA from aligned amino acid sequences. Nucleic Acids Research, 31, 3537–3539. 10.1093/nar/gkg609 12824361 PMC169015

[mec14865-bib-0072] Wybouw, N. , Zhurov, V. , Martel, C. , Bruinsma, K. A. , Hendrickx, F. , Grbić, V. , & Van Leeuwen, T. (2015). Adaptation of a polyphagous herbivore to a novel host plant extensively shapes the transcriptome of herbivore and host. Molecular Ecology, 24, 4647–4663. 10.1111/mec.13330 26211543

[mec14865-bib-0073] Xu, J. , Lin, K. , & Liu, S. S. (2011). Performance on different host plants of an alien and an indigenous *Bemisia tabaci* from China. Journal of Applied Entomology, 135, 771–779. 10.1111/j.1439-0418.2010.01581.x

[mec14865-bib-0074] Yu, Q. Y. , Fang, S. M. , Zhang, Z. , & Jiggins, C. D. (2016). The transcriptome response of *Heliconius melpomene* larvae to a novel host plant. Molecular Ecology, 25, 4850–4865. 10.1111/mec.13826 27572947

[mec14865-bib-0075] Zeng, R. S. , Wen, Z. , Niu, G. , Schuler, M. A. , & Berenbaum, M. R. (2009). Enhanced toxicity and induction of cytochrome P450s suggest a cost of “eavesdropping” in a multitrophic interaction. Journal of Chemical Ecology, 35, 526–532. 10.1007/s10886-009-9640-6 19430966

