# Supplementary material for: Species‐complex diversification and host‐plant associations in Bemisia tabaci: A plant‐defence, detoxification perspective revealed by RNA‐Seq analyses
Source: Mol Ecol. 2018 Oct 10;27(21):4241–56. doi: 10.1111/mec.14865 (PMC6334513; doi:10.1111/mec.14865)
Supplement: Supplementary file 3 [file MEC-27-4241-s003.docx]

***Supplementary Material***

**Table S1**. The *Bemisia tabaci* species complex host range (botanical family and order levels). Literature survey documented field collection surveys of *B. tabaci*, in which the sample collected was genotyped using the mitochondrial cytochrome oxidase I (mtCOI) DNA sequencing method, and the host plant species was recorded. Similar botanical orders share the same background color.


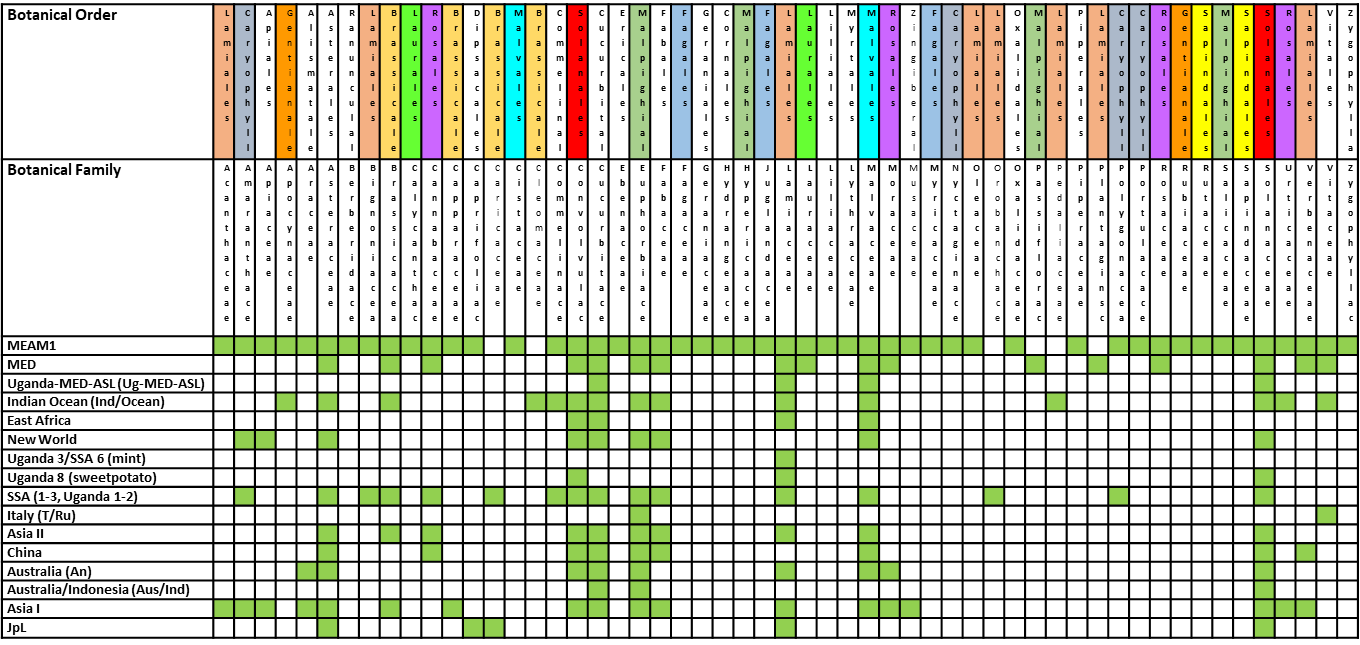


**Data from:** **Ahmed et al.**, J. Econ. Entomol. (2010), 103: 1848-1859; **Alemandri et al**., J. Econ. Entomol. (2012), 105:48-53; **Bayhan et al.**, J. Pest Sci. (2006), 79:233–240; **Brown et al.**, Annu. Rev. Entomol. (1995), 40:511-534; **Charaabi et al.**, J. Entomol. Zool. Stud. (2015), 3: 32-41; **Chowda-Reddy et al.**, J. Integr. Agric. (2012), 11: 235-248; **Costa & Brown**, Vegetable Report (1991); **De Barro et al.**, Aust. J. Entomol. (1998), 37: 214-218; **De Barro**, Mol. Ecol. (2005), 14:3695–3718; **Delatte et al.**, Bull. Entomol. Res. (2005), 95:29–35; **Firdaus et al.**, Insect Sci. (2013), 20:723–733; **Guo et al.**, J. Integr. Agric. (2012), 11:206-214; **Hsieh et al.**, Ann. Entomol. Soc. Am. (2006), 99: 768-775; **Hu** et al., PLoSONE (2011), 6: e16061; **Hu et al.**, J. Insect Sci. (2014), 14:281; **Kijima et al.**, Jpn. J. Appl. Entomol. Zool. (2011), 55: 9–17; **Kijima et al.**, Jpn. J. Appl. Entomol. Zool. (2011), 55: 249–253; **Laarif et al.**, Phytoparasitica (2015), 43:135–150; **Lee et al.**, Fla. Entomol. (2014), 97:155-161; **Manzari & Quicke**, J. Nat. Hist. (2006), 40:44-46, 2423-2554; **Marubayashi** **et al.**, J. Appl. Entomol. (2013), 137:113–121; **Muniz et al.**, Environ. Entomol. (2011), 40:989-998; **Parrella et al.**, J. Pest Sci. (2012), 85:227–238; **Qin et al.**, Insect Sci. (2016), 23: 215-224; **Roopa et al.**, Fla. Entomol. (2015), 98: 1062-1071; **Ruihua et al.**, Plant Dis. Pest. (2016), 7: 1-4; **Simmons et al.**, Ann. Entomol. Soc. Am. (2000), 93: 856-861; **Sseruwagi et al.**, Ann. Appl. Biol. (2005), 147:253–265; **Sseruwagi et al.**, Entomol. Exp. Appl. (2006), 119:145–153; **Tahiri** **et al.**, Ann. Appl. Biol. (2006), 149:175–186; **Tajebe et al.**, J. Appl. Entomol. (2015), 139:627–637; **Tajebe et al.**, Ann. Appl. Biol. (2015), 166:297–310; **Ueda et al.**, J. Appl. Entomol. (2009), 133:355–366; **Zheng**, Phytoparasitica (2006), 34:431-440.

**Table S2.** The Bta number or accession number in the *Bemisia tabaci* MEAM1 genome database (Chen et al., 2016) for genes reported in Figure 4 (genes significantly overexpressed or underexpressed in one species compared to all others) and Figure 6 (genes that were found to be plastically-expressed in more than one species and showing the same expression pattern in at least two species), found in an Excel file, “Supplementary Table S2.xlsx”. Table S2 also provides, for each listed gene, the accession numbers of the best BLAST hit (e-value threshold 10−6), along with a putative function, in the insect model species: *Drosophila melanogaster*, *Helicoverpa armigera* and *Acyrthosiphon pisum*. Full sequences are provided for genes where conflicts were found between our database and the *Bemisia tabaci* MEAM1 genome database (Chen et al., 2016) or when the full-sequence was missing from the latter.

**Table S3.** Primers and qRT-PCR protocol. Primers were designed using the NCBI’s Primer-Blast tool (https://www.ncbi.nlm.nih.gov/tools/primer-blast/), based on cDNA sequences retrieved from the RNA-seq. Specificity was improved using the Primersearch tool of EMBOSS (Rice, Longden, & Bleasby, 2000) implemented in Galaxy, allowing 30% mismatches. Only primer pairs with a sole target sequence were utilized. The qRT-PCRs were performed using as template new RNA samples, extracted from the MEAM1 and SSA-SG3 populations feeding on eggplant. An aliquot of 500 ng total RNA from each of the three biological replicates, for each species, served as a template for cDNA synthesis with 5X All-In-One RT MasterMix (Applied Biological Materials), according to the manufacturer’s instructions. PCR reactions of 18 µl contained 9 µl Power SYBR Green PCR Master Mix (Applied Biosystems), 150 nM forward and reverse primers and 2 µl of cDNA. PCR thermal conditions consisted one cycle of 95 ^o^C for 1 min, followed by 40 cycles of 95 ^o^C for 15 sec and 60 ^o^C for 1 min. Melt curve analyses were performed to test the specificity of amplicons. A serial dilution of cDNA was used to generate standard curves for each gene in order to assess the PCR efficiency and quantitative differences amongst samples. Quantification of the transcript level was conducted according to the ∆CT method using the ribosomal protein L13a (RPL13A) as the reference gene (Collins et al., 2014). qRT-PCR’s Primers with Standard Curve details (R^2^ and slope) and the product sizes are presented below.

| **gene** | **primer sequence 5'->3'** | **product size** | **MEAM1** | | **SSA1-SG3** | |
| --- | --- | --- | --- | --- | --- | --- |
|  |  |  | **slope** | **R^2^** | **slope** | **R^2^** |
| *UDP_15* | TGATTACTACCTCGTTTCCGG | 103 | -3.25 | 0.98 | -3.31 | 0.98 |
|  | ACCCATCCAGCAGTTTTTCA |  |  |  |  |  |
| *UDP_28* | CGGTTCTGTTCATGACCCAT | 97 | -3.2 | 0.98 | -3.28 | 0.98 |
|  | AATTGATCCACGATCACGGG |  |  |  |  |  |
| *UDP_55* | GCTGAGGAATGTTTCGCTTT | 88 | -3.54 | 0.98 | -3.3 | 0.98 |
|  | CTCAATCACATTCGGTGGGA |  |  |  |  |  |
| *UDP_63* | CCAGTATTTTCACGAGAGCCT | 96 | -3.36 | 0.98 | -3.48 | 0.97 |
|  | TGCCCACACTTCCAAAAATG |  |  |  |  |  |
| *GST_25* | GGCACCCTTTATCAGGCTAT | 142 | -3.29 | 0.98 | -3.18 | 0.97 |
|  | CCTGCCGCAAATTTCGATAC |  |  |  |  |  |
| *GST_28* | GCGGCTATTTAGTGAACGGA | 125 | -3.48 | 0.99 | -3.35 | 0.99 |
|  | CCTTCAGGGCTATGATAAGAGG |  |  |  |  |  |
| *P450_41* | TCCTTCCTATCAGCTGGCTT | 125 | -3.5 | 0.987 | -3.44 | 0.98 |
|  | GGATTCTTTTCTGACCGCCT |  |  |  |  |  |
| *P450_79* | AGAGTGCTGAAACAGTCGAG | 121 | -3.29 | 0.97 | -3.11 | 0.98 |
|  | GTTTGACCTTGCTCGTTGAC |  |  |  |  |  |
| *ABC_26* | TAAACCAGCAGAAGGAACGC | 134 | -3.41 | 0.98 | -3.36 | 0.979 |
|  | GATCCTCTTTCACTGGCACT |  |  |  |  |  |
| *RPL13A* | CATTCCACTACAGAGCTCCA | 100 | -3.55 | 0.976 | -3.42 | 0.98 |
|  | TTTCAGGTTTCGGATGGCTT |  |  |  |  |  |

**Table S4.** Comparison between the RNA-seq and qRT-PCR analyses. Quantification of the transcript level was conducted according to the ∆CT method using the ribosomal protein L13a (RPL13A) as the reference gene (Collins et al., 2014). Comparisons of transcription levels were conducted using a one-way ANOVA model (species, MEAM1 versus SSA1-SG3, as the main effect) for each gene separately. Genes were considered significantly over- or under-transcribed when the ∆CT values of RNA samples from MEAM1 were different from ∆CT values of RNA samples from SSA1-SG3 at *P* ≤ 0.05. All statistical analyses conducted were performed with JMP statistical software version 13 (SAS Institute, USA).

| **Gene** | **Expected from RNA-seq** | **Verification by qRT-PCR** | **∆∆CT (MEAM1 - SSA1-SG3)^a^** | **Significance of differences between ∆CT values** | **Log_2_FC (MEAM1 - SSA1-SG3)** | **Significance of differences between Log2FC values** |
| --- | --- | --- | --- | --- | --- | --- |
| *UDP_15* | SSA1-SG3 > MEAM1 | SSA1-SG3 > MEAM1 | 3.36 | <.0001 | -1.61 | 0.0003 |
| *UDP_28* | SSA1-SG3 < MEAM1 | SSA1-SG3 < MEAM1 | -1.56 | 0.0306 | 2.27 | <.0001 |
| *UDP_55* | SSA1-SG3 > MEAM1 | SSA1-SG3 > MEAM1 | 1.73 | <.0001 | -1.93 | <.0001 |
| *UDP_63* | SSA1-SG3 > MEAM1 | SSA1-SG3 > MEAM1 | 2.73 | <.0001 | -2.41 | <.0001 |
| *GST_25* | SSA1-SG3 < MEAM1 | SSA1-SG3 < MEAM1 | -3.69 | <.0001 | 5.30 | <.0001 |
| *GST_28* | SSA1-SG3 ≅ MEAM1 | SSA1-SG3 ≅ MEAM1 | 0.35 | 0.1972 | -0.012 | 0.9963 |
| *P450_41* | SSA1-SG3 > MEAM1 | SSA1-SG3 > MEAM1 | 3.08 | <.0001 | -3.12 | <.0001 |
| *P450_79* | SSA1-SG3 > MEAM1 | SSA1-SG3 > MEAM1 | 2.6 | <.0001 | -2.03 | <.0001 |
| *ABC_26* | SSA1-SG3 > MEAM1 | SSA1-SG3 > MEAM1 | 1.33 | <.0001 | -1.40 | <.0001 |

^a^ Be aware that positive ∆∆CT value indicates that the ∆CT value in MEAM1 is higher than in SSA1-SG3 and vice versa. ∆CT values are inverse to the amount of targeted mRNA in the sample. Lower ∆CT values indicate high amounts of targeted mRNA, while higher ∆CT values mean lower amounts of targeted mRNA.

**Table S5.** Maximum Likelihood estimates of the ancestral state reconstruction at host plants order, family and genus (*Solanum* and *Capsicum* genera only) levels, found in an Excel file, “Supplementary Table S5.xlsx”.

**Figure S1.** Maximum Likelihood (ML) ancestral host-plant usage reconstruction for all major genetic groups of *B. tabaci* and two related outgroups (*Bemisia afer* and *Dialeurodes citri*). The ML inferred tree (**a**) was produced using selected mtCOI nucleotide sequences. Ultrafast bootstrap values are shown at each node. The host plant ranges of the last common ancestors (LCAs) of the different *B. tabaci* species (**b-e**) were estimated with the ace function in the ape package of R using the information from Table S1. Paraphyletic branches were forced to be monophyletic. Circles at the tips represent the ability of the specific species not to exploit (blue) or to exploit (other colors) the order/family/genus analyzed. Pie charts at nodes represent the probability (0-1) of the LCA to exploit (other colors) or not exploit (blue) the order/family/genus analyzed. LCA2 was considered the last common ancestor of the *B. tabaci* species complex.


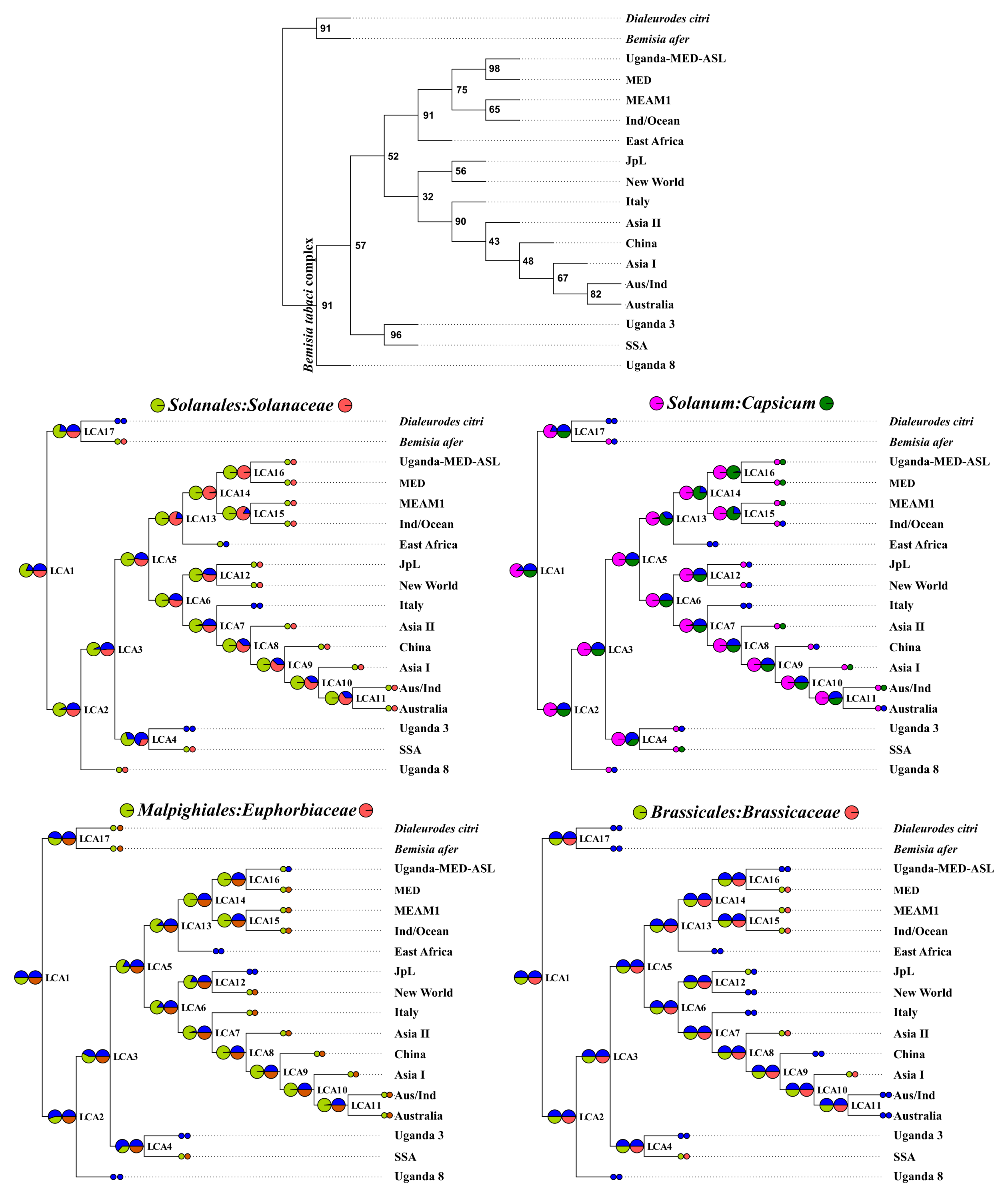


**Figure S2.** *De novo* transcriptomes-assemblies nucleotide identity. We used a “blast reciprocal best hit” approach to check the identity of each gene/contig (of the relevant detoxification gene) in the species’ transcriptome to its putative orthologous gene/contig in the manually curated dataset (represented by each dot). All shown alignments include genes/contigs that had at least 70% of their sequence aligned with a cutoff of at least 50% identity. The number of transcripts analyzed between species vary because it was not always possible to recover the best orthologous transcript (e.g. genes were not expressed or were highly fragmented). Errors bars represent standard error of the means.


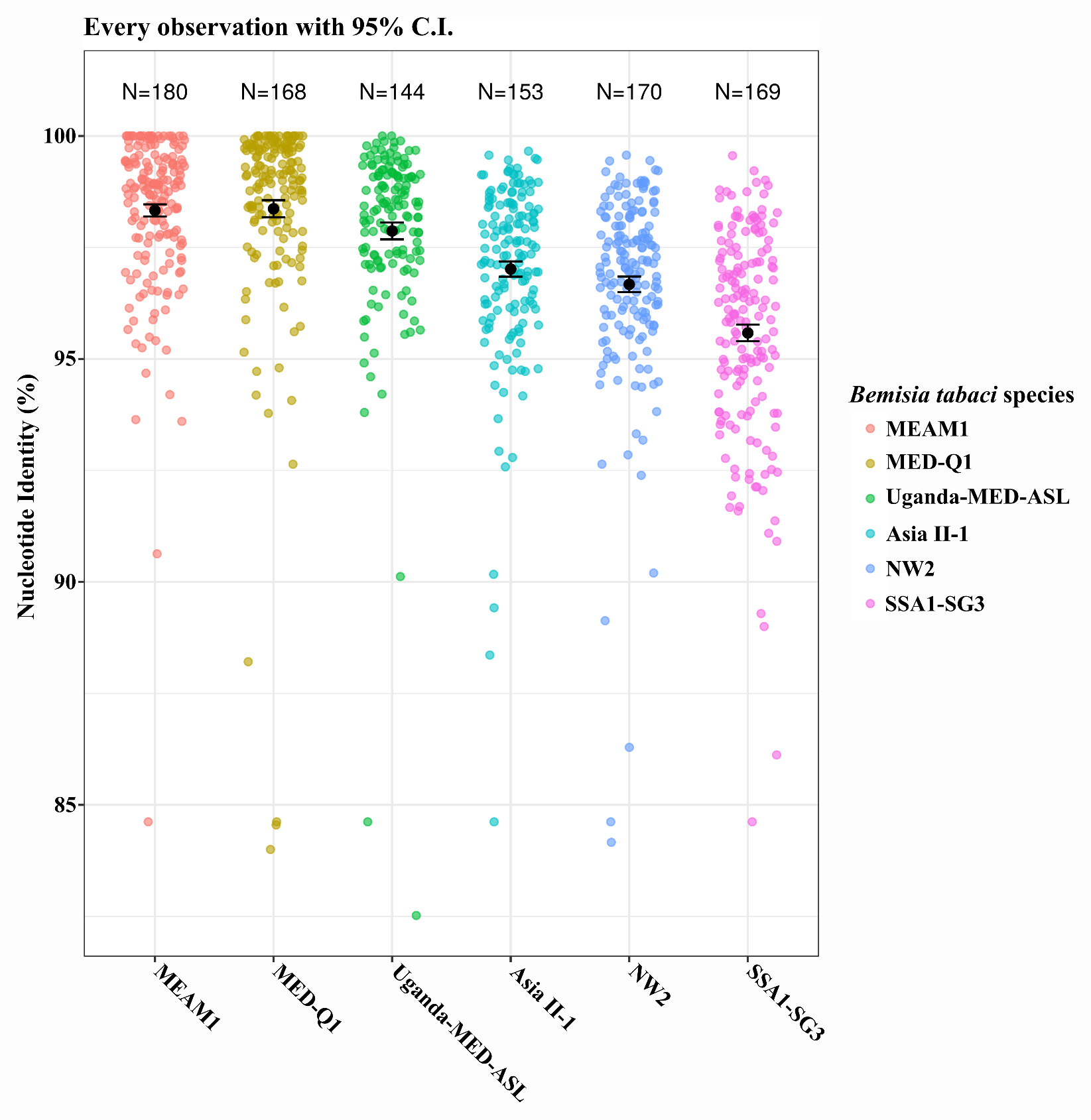


**Figure S3.** Maximum Parsimony ancestral host-plant usage reconstruction for all major genetic groups of *B. tabaci* and two related outgroups (*Bemisia afer* and *Dialeurodes citri*). Analysis focus on nine orders (belonging to the Asterids and Rosids clades) that are commonly shared by most *B. tabaci* species. The same ML inferred tree (Fig. S1a) was utilized here (see bootstrapping values in Fig.S1a). The host plant ranges of the last common ancestors (LCAs) of the different *B. tabaci* species were estimated with the MPR function (using an unrooted tree) in the ape package from R using the information from Table S1. LCA2 was again considered the last common ancestor of the *B. tabaci* species complex. The black triangle denotes the mid-point of the unrooted tree. Blue circles at the tips denote a non-used plant host order by the specific species while yellow circles denote usage of the plant host order by the specific species. Triangles at nodes represent the ability of the LCA to exploit (yellow) or not exploit (blue) the order analyzed. Red triangles stand for undetermined (“unknown”) associations.


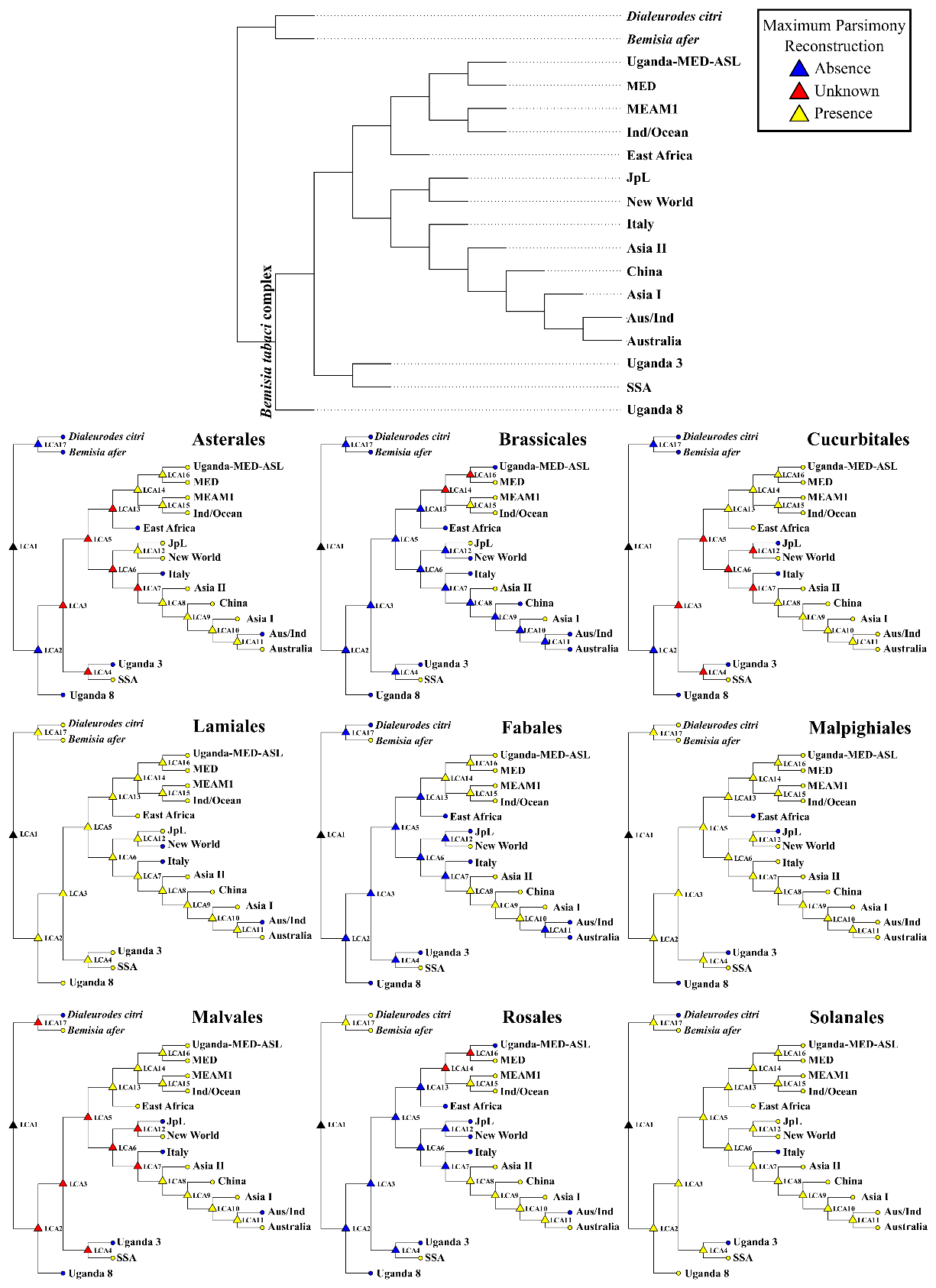


**Figure S4**. Principal components analysis was performed on the data from the 266 differentially expressed genes across the 24 insect species — plant host combinations. The graph shows principal component 1 vs. principal component 2 and 3 values for each combination (three biological replicates) in this study.

**
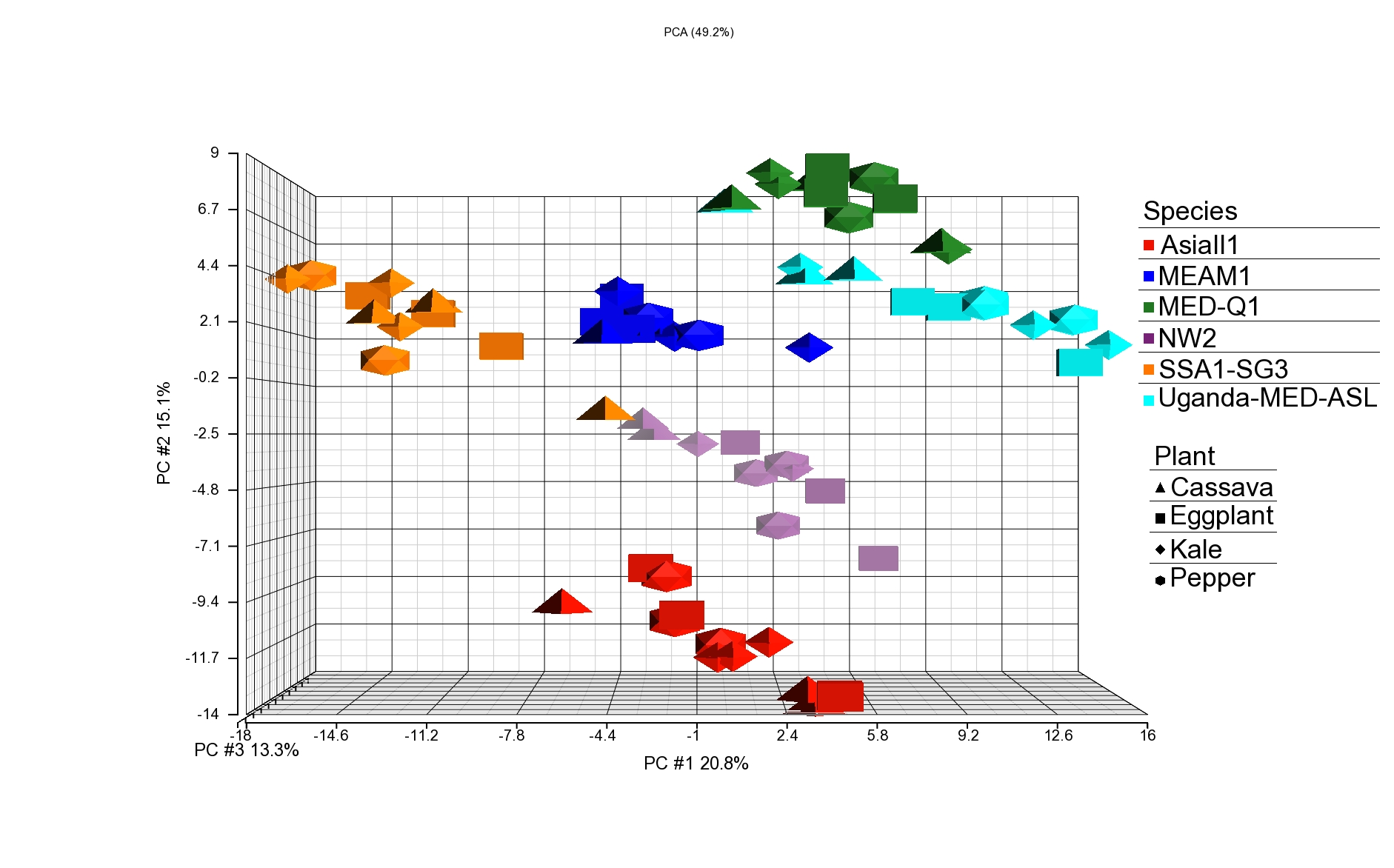
**

**References**

Chen, W., Hasegawa, D. K., Kaur, N., Kliot, A., Pinheiro, P. V., Luan, J., ... & Xu, Y. (2016). The draft genome of whitefly *Bemisia tabaci* MEAM1, a global crop pest, provides novel insights into virus transmission, host adaptation, and insecticide resistance. BMC Biology, 14, 110.

Collins, C., Patel, V. B., Colvin, J., Bailey, D., & Seal, S. (2014) Identification and evaluation of suitable reference genes for gene expression studies in the whitefly *Bemisia tabaci* (Asia I) by reverse transcription quantitative real-time PCR. *Journal of Insect Science, 14*, 63.

Rice, P., Longden, I., & Bleasby, A. (2000). EMBOSS: the European Molecular Biology Open Software Suite. *Trends in Genetics, 16*, 16276–16277.
